# Rational drug combination design in patient-derived avatars reveals effective inhibition of hepatocellular carcinoma with proteasome and CDK inhibitors

**DOI:** 10.1186/s13046-022-02436-9

**Published:** 2022-08-15

**Authors:** Jhin Jieh Lim, Lissa Hooi, Yock Young Dan, Glenn K. Bonney, Lei Zhou, Pierce K.-H. Chow, Cheng Ean Chee, Tan Boon Toh, Edward K.-H. Chow

**Affiliations:** 1 Cancer Science Institute of Singapore, National University of Singapore, 14 Medical Drive, 117599 Singapore, Singapore; 2 NUS Centre for Cancer Research (N2CR), Yong Loo Lin School of Medicine, National University of Singapore, Singapore, Singapore; 3 Department of Medicine, Yong Loo Lin School of Medicine, National University of Singapore, Singapore, Singapore; 4 Division of Hepatobiliary and Liver Transplantation Surgery, National University Health System, Singapore, Singapore; 5 Department of Hepatopancreatobiliary and Transplant Surgery, Singapore General Hospital, Singapore, Singapore; 6 Department of Haematology-Oncology, National University Cancer Institute, Singapore, Singapore; 7 The N.1 Institute for Health, National University of Singapore, Singapore, Singapore; 8 The Institute for Digital Medicine (WisDM), Yong Loo Lin School of Medicine, National University of Singapore, Singapore, Singapore; 9 Department of Biomedical Engineering, National University of Singapore, Singapore, Singapore; 10 Department of Pharmacology, Yong Loo Lin School of Medicine, National University of Singapore, Singapore, Singapore

**Keywords:** Combination therapy, Patient-derived xenograft, Organoids, Proteasome inhibitors, CDK inhibitors, Hepatocellular carcinoma

## Abstract

**Background:**

Hepatocellular carcinoma (HCC) remains difficult to treat due to limited effective treatment options. While the proteasome inhibitor bortezomib has shown promising preclinical activity in HCC, clinical trials of bortezomib showed no advantage over the standard-of-care treatment sorafenib, highlighting the need for more clinically relevant therapeutic strategies. Here, we propose that rational drug combination design and validation in patient-derived HCC avatar models such as patient-derived xenografts (PDXs) and organoids can improve proteasome inhibitor-based therapeutic efficacy and clinical potential.

**Methods:**

HCC PDXs and the corresponding PDX-derived organoids (PDXOs) were generated from primary patient samples for drug screening and efficacy studies. To identify effective proteasome inhibitor-based drug combinations, we applied a hybrid experimental-computational approach, Quadratic Phenotypic Optimization Platform (QPOP) on a pool of nine drugs comprising proteasome inhibitors, kinase inhibitors and chemotherapy agents. QPOP utilizes small experimental drug response datasets to accurately identify globally optimal drug combinations.

**Results:**

Preliminary drug screening highlighted the increased susceptibility of HCC PDXOs towards proteasome inhibitors. Through QPOP, the combination of second-generation proteasome inhibitor ixazomib (Ixa) and CDK inhibitor dinaciclib (Dina) was identified to be effective against HCC. In vitro and in vivo studies demonstrated the synergistic pro-apoptotic and anti-proliferative activity of Ixa + Dina against HCC PDXs and PDXOs. Furthermore, Ixa + Dina outperformed sorafenib in mitigating tumor formation in mice. Mechanistically, increased activation of JNK signaling mediates the combined anti-tumor effects of Ixa + Dina in HCC tumor cells.

**Conclusions:**

Rational drug combination design in patient-derived avatars highlights the therapeutic potential of proteasome and CDK inhibitors and represents a feasible approach towards developing more clinically relevant treatment strategies for HCC.

**Supplementary Information:**

The online version contains supplementary material available at 10.1186/s13046-022-02436-9.

## Background

Hepatocellular carcinoma (HCC) is the most common malignancy of the liver and the third most common cause of cancer-related deaths worldwide [1]. A significant challenge in the management of the disease is the lack of effective treatment options, especially for patients with advanced and unresectable HCC. Sorafenib, an oral multi-kinase inhibitor, was the first drug to be approved by the FDA for the treatment of advanced HCC in 2007 and remains one of the standard-of-care treatments in addition to the recently approved combination of atezolizumab and bevacizumab in the first line setting [2, 3]. While several new targeted therapies such as regorafenib, lenvatinib, cabozantinib and ramucirumab [4–7] have recently been approved, survival benefits remain modest, and many of these compounds are used as second-line treatments for sorafenib-refractory patients [8, 9]. The dearth of effective treatment options available for advanced HCC necessitates continued investigations into new treatment approaches and therapeutic targets.

Proteasome inhibitors are a promising class of compounds that mitigate the tumor cell’s ability to overcome the increase in side products of protein synthesis, such as the accumulation of misfolded proteins, thus leading to cell death [10]. Bortezomib is a first-in-class proteasome inhibitor that was approved by the FDA for the treatment of multiple myeloma and mantle cell lymphoma [11–13]. Due the development of bortezomib resistance and off-target toxicities [14–17], second-generation proteasome inhibitors, including carfilzomib and ixazomib, were subsequently developed with higher potencies, reduced side effects and have also been approved for multiple myeloma treatment [18–20]. In light of their success in hematological malignancies, proteasome inhibitors have been evaluated in various solid tumors, including HCC [21–23]. In preclinical studies using HCC cell lines, proteasome inhibitors have been reported to induce various anti-tumor responses, such as cell cycle arrest, apoptosis, induction of endoplasmic reticulum (ER) stress, repression of NFκB signaling, and inhibition of epithelial-mesenchymal transition [24, 25]. However, a phase 2 trial in HCC showed that bortezomib lacked significant single-agent activity compared to sorafenib despite its promising preclinical results [23]. Another phase 2 study evaluating bortezomib in combination with doxorubicin also reported inferior survival outcomes when compared to sorafenib [26], highlighting the lack of success in translation of preclinical results to clinical efficacy. This unsuccessful translation has also been observed in many other clinical trials of drug combinations in HCC [27–29], and could be the result of disease heterogeneity and poorly designed drug combinations arising from a lack of understanding of the key mechanisms driving HCC tumor progression [30].

Given that combination therapy design in HCC is often based on historical treatment options or mechanisms derived from monocultured commercial cell line models, advancements in combination therapies have been modest and limited. The preclinical efficacy of proteasome inhibitors likely reflects a therapeutic vulnerability that can be exploited through better treatment strategies, such as in combination with the right drugs. Thus, we propose a more rational approach in identifying efficacious proteasome inhibitor-based drug combinations by pairing a hybrid experimental-computational platform called Quadratic Phenotypic Optimization Platform (QPOP) with more clinically relevant patient-derived models of HCC.

QPOP uses the least number of experimental test points to interrogate the entire drug-dose search space in the search for optimal drug combinations [31]. The fundamental basis of QPOP, and similar approaches, is the discovery that biological response can be mapped to a quadratic regression where higher order terms do not have significant impact [31–35]. As such, quantifiable biological responses, such as cancer cell death, can be mapped towards interactions within a set of inputs, such as drugs, in order to identify the most effective combination of inputs for a desired biological response. QPOP has been shown to successfully identify novel drug combinations that outperform standard clinical treatments in drug-resistant hematological cancers [31, 36]. Furthermore, when QPOP was applied on patient-derived materials, QPOP-predicted drug combinations were clinically effective against a lymphoma case that had failed multiple lines of therapy [36]. These previous studies suggest that drug combinations identified by QPOP using patient-derived HCC models could be clinically effective.

Thus, in this study, we evaluated these drug combinations in a cohort of human HCC patient-derived xenograft (PDX) and PDX-derived organoid (PDXO) models, which can better recapitulate disease heterogeneity and molecular profiles of primary patient tumors [37–40], to achieve more accurate tumor drug responses. We postulated that investigating these drug combinations in the context of HCC molecular heterogeneity could identify proteasome inhibitor-based drug combinations that are more likely to be effective in the clinical setting.

## Methods

### Animals

All animal experiments were performed according to guidelines and protocols approved by the National University of Singapore Institutional Animal Care and Use Committee (IACUC). Female NOD/SCID/IL2rγ^null^ (NSG) mice of 6- to 8-week-old were purchased from InVivos, Singapore, and kept on a Teklad global 18% protein rodent diet in a 12-hour light-dark cycle regulated environment.

### Generation of patient-derived xenografts (PDXs) and organoid (PDXO) cultures

The collection of HCC patient samples, generation and use of PDXs were performed under domain-specific review board protocol approval by the National Healthcare Group Domain-Specific Review Board as well as human biomedical research protocol approval by the NUS-Institutional Review Board. Primary HCC samples were obtained from the National University Hospital and Singapore General Hospital during liver resection with prior written informed consent from patients, and diagnosis was confirmed by histology. Tissues were stored in tissue storage solution (Miltenyi Biotec) prior to processing for implantation into NSG mice. Briefly, tumor tissues were minced into small pieces and mixed with Matrigel (Corning) in a 1:1 ratio (v/v) to achieve a final volume of 200 μL per injection. For each patient tumor, the tissue-Matrigel mixtures were injected into flanks of at least 2 NSG mice. PDX tumor growth was monitored by measuring tumor diameter twice a week. Tumors were harvested for serial transplantation into successive NSG mice upon reaching 15 mm in diameter, using the same methods as described above. To date, up to 35 HCC PDX models/lines have been successfully established at Cancer Science Institute of Singapore, NUS and have been used to create PDX-derived organoid (PDXO) lines, of which 18 were used in this study.

For HCC PDXO isolation and culture, PDX tumor tissues were first harvested and finely minced before incubation with 1 mg/mL collagenase/dispase (Roche) for 10 mins at 37 °C. The tumor suspension was then washed with Dulbecco’s Modified Eagle Medium (DMEM) (Biowest) and filtered through 100 μm cell strainer, followed by centrifugation for 5 mins at 1000 rpm. Tumor cells were resuspended in 1X red blood cell lysis buffer (Invitrogen) and incubated on ice for 3 mins, before washing and centrifuging for 5 mins. Cells were subsequently counted and seeded into cell culture flasks in DMEM/F12 medium (Biowest) supplemented with defined growth factors [38, 41] for culture and further experiments.

### In vivo drug treatment study

Dissociated PDX tumor cells (100,000 cells) were injected into the flanks of 6- to 8-week-old female NSG mice. The mice were then randomized into 5 treatment groups (*n* = 5 to 9 per group), which include vehicle control, ixazomib (7 mg/kg), dinaciclib (5 mg/kg), Ixa + Dina, and sorafenib (30 mg/kg). After 1 week, the mice were administered with their respective treatments three times a week by intraperitoneal injection (dinaciclib) and oral gavage (ixazomib, sorafenib). All drugs were purchased from Selleck Chem. Tumor growth was assessed by measuring tumor volume, which can be defined as V = π/6 x A^2^ x B, where A is the smallest superficial diameter and B is the largest superficial diameter. All mice were terminated when tumors in the vehicle control group reached endpoint, defined by a tumor volume of 2000 mm^3^.

### Cell culture

Human liver epithelial cell line THLE-2 was obtained from American Type Culture Collection (ATCC, CRL2706) and cultured in Bronchial Epithelial Cell Growth Medium (BEGM) complete medium (Lonza), which comprises Bronchial Epithelial Cell Growth Basal Medium, BEGM Bullet Kit supplements and growth factors, and 10% FBS. All cells were maintained at 37 °C in a humidified atmosphere with 5% CO_2_.

### Drug library screen and serial dose-response assays

HCC PDXOs were seeded in 384-well plates at a density of 2000 cells/well 1 day before treatment with drugs from the Discovery Probe FDA-approved drug library (ApexBio, L1021) at 1 μM concentration. After 48 hours, cell viability was measured using CellTiter-Glo Luminescent Cell Viability Assay (Promega, G5771) as per manufacturer’s instructions. For dose-response assays, cells were also seeded in 384-well plates (2000 cells/well) overnight prior to treatment with selected drugs at concentrations ranging from 0.0001 μM to 100 μM (*n* = 5 per treatment). Cell viability was measured 48 hours after treatment as described above, and dose-response curves were generated using Prism 8 software (GraphPad) to compute respective IC_50_ values.

### QPOP combinatorial treatment and analysis

HCC PDXOs and THLE-2 cells were seeded at 2000 cells/well density in 384-well plates 1 day before drug treatment. The drug combinations used for QPOP for nine drugs at three dosage levels were generated using an orthogonal array composite design (OACD) as described by Xu et al. [42], which combines a three-level orthogonal array with a two-level fractional factorial design to achieve the least number of combinations for factor screening. The three dosage levels used are IC_0_, IC_10_, and IC_20_, which were determined based on the dose-response curves of each drug across the panel of HCC PDXOs. Drug combinations were prepared using an automated liquid handler (Mini Janus, Perkin Elmer), and after 48 hours of drug treatment, cell viability was measured using CellTiter-Glo Luminescent Cell Viability Assay (Promega, G5771) as per manufacturer’s instructions. QPOP analysis was performed as previously described [31], using the viabilities of THLE-2 (viability_THLE2_) and HCC PDXOs (viability_PDXO_) as readouts. Briefly, the viability for each experimental combination was used as data points to fit a second-order quadratic series that generates all possible drug combinations with their corresponding projected output values. All two-drug combinations were subsequently ranked according to the output and used for hierarchical clustering and frequency analysis.

### Drug combination validation

Validation of drug combinations was performed using single-drug and combination dose-response assays to calculate the combination index (CI) and dose reduction index (DRI) values based on the Chou-Talalay method [43]. Briefly, cells were seeded in 384-well plates at a density of 2000 cells/well 1 day before drug treatment for 48 hours. Cell viability was then measured as described above. For the combination dose-response assay, a fixed dose ratio between ixazomib and dinaciclib (as derived from QPOP analysis) was used.

### Viability and proliferation assays

To determine viability and proliferation of HCC PDXOs in culture, dissociated PDXOs were seeded into 24-well plates and monitored over a period of 8 days. The number of viable cells were counted using Trypan Blue staining after 2-, 4-, 6- and 8-days post-seeding. For drug treatment viability assays, PDXOs were treated with ixazomib (Selleck Chem) at 0.1 μM and dinaciclib (Selleck Chem) at 0.01 μM individually and in combination for 48 hours prior to viability measurement with CellTiter-Glo (Promega, G7571).

### Immunohistochemistry and immunofluorescence

PDX tumor tissues were fixed in 4% paraformaldehyde immediately after extraction and embedded in paraffin. Immunohistochemical staining was performed on tissue sections of 4 μm thickness using standard procedures with the following antibodies: c-Jun (Cell Signaling), Ki67 (Abcam). Images were acquired using Vectra® imaging system (Akoya Biosciences) and analyzed using inForm® software (Akoya Biosciences). At least 10 fields (200X magnification) were randomly chosen for each sample for DAB quantification.

For multiplex immunofluorescence staining, drug-treated HCC PDXOs were first adhered to glass slides using cytospin and fixed in 4% formaldehyde before permeabilization with 0.1% Triton X-100. The Opal™ (Akoya Biosciences) workflow was then used to serially stain slides with c-Jun and cleaved caspase 3 antibodies (Cell Signaling). Briefly, slides were incubated with c-Jun primary antibody overnight followed by HRP-conjugated secondary antibody and tyramide signal amplification (TSA) with Opal 520 fluorophore. Slides were subsequently stained with cleaved caspase 3 for 1 hour before addition of HRP and TSA reagent with Opal 690 fluorophore. DAPI was added last as a nuclear counterstain. Images were acquired using Vectra® imaging system (Akoya Biosciences) and analyzed using inForm® software (Akoya Biosciences). For quantification, 10 fields (200X magnification) were randomly selected for each sample. Nuclei and cytoplasmic fluorophore-specific intensities were assessed, and positive expression was determined using the median intensities (calculated from all stained samples) as thresholds.

For detection of HCC markers in matched PDXs and PDXOs, primary antibodies for HepPar1 (NovusBio), AFP, CK19 and GPC3 (Abcam) were used for fluorescent immunohistochemical staining on PDX tumor tissues and immunofluorescence staining on PDXOs. Overnight incubation with the primary antibodies was followed by application of HRP-conjugated secondary antibody and TSA with Opal 520 fluorophore (Akoya Biosciences) for PDX tissues, and secondary antibody conjugated with Alexa Fluor 488 (Invitrogen) for PDXOs. DAPI was added as a nuclear counterstain. Images were acquired using Vectra® imaging system (Akoya Biosciences). Further details on the primary antibodies used are listed in Supplementary Table S1.

Aggresome staining was performed on treated PDXOs using PROTEOSTAT Aggresome Detection Kit (Enzo Life Sciences, ENZ-51035) as per manufacturer’s instructions. Slides were imaged using Vectra® imaging system and cytoplasmic aggresome staining was similarly quantified as described above.

### Immunoblot analysis

Cells were lysed in buffer containing 0.5% sodium deoxycholate, 1% NP-40 detergent, 0.1% SDS, 0.15 M NaCl, 10 mM Tris-HCl pH 7.4, with the addition of protease and phosphatase inhibitor cocktail tablets (Roche). Equal amounts of protein lysates were resolved by SDS-PAGE and transferred onto PVDF membranes according to standard procedures. Membranes were stained with primary antibodies followed by HRP-linked secondary antibodies before chemiluminescent detection using the ChemiDoc imaging system. Primary antibodies used for immunoblot analyses include CHOP, GRP78, cleaved caspase 3, caspase 3, PARP, phospho-c-Jun (Ser63), c-Jun, phospho-JNK (Thr183/Tyr185), JNK, phospho-Rb (Ser807/811), CDK1, CDK2, CDK9, NFκB1, phospho-ERK (Thr202/Tyr204), ERK, phospho-p38 (Thr180/Tyr182), p38 (Cell Signaling Technology); ATF4 (Santa Cruz); CDK5 (Invitrogen); β-actin (Sigma). Additional details of the antibodies used are included in Supplementary Table S1.

### Apoptosis assays

Cells were treated with drugs or DMSO control for 24 hours prior to apoptosis staining using FITC Annexin V antibody and Propidium Iodide (PI) as per manufacturer’s instructions (BD Biosciences, 556547). Apoptosis was quantified using flow cytometry (BD LSRII, BD Biosciences) and analyzed using FlowJo software.

To determine apoptosis in HCC PDX tumors, terminal deoxynucleotidyl transferase-mediated dUTP nick-end labeling (TUNEL) assay was performed using ApopTag® Fluorescein In Situ Apoptosis Detection Kit (Millipore, S7110) according to manufacturer’s instructions. Tissues were counterstained with DAPI before image acquisition using the Vectra® imaging system (Akoya Biosciences). Quantification of TUNEL-positive cells was analyzed using inForm® software (Akoya Biosciences).

### Cell cycle analysis

Following 24-hour drug treatment, PDXOs were harvested and fixed with 70% ethanol overnight before incubation with RNAse A (100 μg/ml) and propidium iodide (50 μg/ml) for 10 mins. Cell cycle distribution was measured using flow cytometry (BD LSRII, BD Biosciences) and analyzed using FlowJo software.

### Proteasome activity assay

Cells were seeded at 5000 cells/well density in 96-well plates 1 day before drug treatment. Proteasomal chymotrypsin activity was measured at 4 hours and 24 hours post-treatment using Proteasome-Glo Chymotrypsin-Like Cell-Based Assay (Promega, G8660) as per manufacturer’s instructions.

### RNA isolation and quantitative RT-PCR

After 48 hours of drug treatment, HCC PDXOs were harvested and subjected to RNA isolation using RNeasy kit (Qiagen, 74104). Cellular RNA was then reverse transcribed into cDNA using the iScript Reverse Transcription Supermix (Bio-Rad Laboratories, 1708841) according to manufacturer’s instructions. For quantitative real-time PCR analysis, each sample was measured in triplicate using iTaq Universal SYBR Green Supermix (Bio-Rad Laboratories, 1725121) on a QuantStudio 3 Real-Time PCR system (Applied Biosystems). Primers used are as follows: *GADD45B* (forward primer, 5′-GACAACGACATCAACATCGTGC-3′; reverse primer, 5′-CGTGACCAGGAGACAATGC-3′), *JUN* (forward primer, 5′-GAGCTGGAGCGCCTGATAAT-3′; reverse primer, 5′-CCCTCCTGCTCATCTGTCAC-3′), and *GAPDH* (forward primer, 5′-AAGGTGAAGGTCGGAGTCAA-3′; reverse primer, 5′-AATGAAGGGGTCATTGATGG-3′). All mRNA levels were normalized to GAPDH and expressed as mean ± SD.

### NanoString gene expression analysis

100 ng of extracted RNA from PDXOs were subjected to multiplex gene expression analyses using nCounter PanCancer Pathways Panel (NanoString Technologies) on the NanoString nCounter SPRINT profiler system as per manufacturer’s instructions. A total of 770 genes (730 cancer-related human genes and 40 internal reference genes) were evaluated. Results were analyzed using nSolver Analysis and nCounter Advanced Analysis software (NanoString Technologies), in which gene expression levels were normalized to the in-built set of positive and negative control genes [44]. Differential expression of key transcriptomic pathways was compared between the treatment groups (Ixazomib, Dinaciclib, Ixa + Dina) and DMSO control, with fold change and *P* values calculated based on nSolver’s recommended default settings. A Venn diagram was constructed to determine overlapping genes with a fold change of ≥ ±2 between PDXO1, 11 and 12. Gene set analysis and global significance scores were calculated from differential expression analysis-derived t-statistics for genes in a particular gene set or pathway, as defined by the software.

### Inhibition of JNK

PDXOs were transfected with siRNAs against *MAPK8* and *JUN* (TriFECTa® DsiRNA Kit, Integrated DNA Technologies, IDT, Singapore) using Lipofectamine RNAiMAX (Thermo Fisher), or treated with JNK inhibitor SP600125 (Selleck Chem) and incubated overnight prior to treatment with ixazomib and dinaciclib for 24 hours. Cells were then pelleted and used for RNA and protein extraction as described above.

### Statistical analysis

All experiments were performed in at least three replicates, with data presented as mean ± standard deviation (SD), unless otherwise stated. For QPOP analysis, parameter estimation to obtain correlation coefficients was achieved using sum of squares F-test, and the predictive power of QPOP generated outputs was determined via adjusted R^2^ value. Unpaired two-tailed Student’s *t* test was used for statistical comparisons between two independent groups. Kruskal-Wallis test was used to compare each proteasome inhibitor with other clinical drugs in the FDA drug screen. Pearson correlation coefficient was used to determine the relationship between pathway expression and drug sensitivity. *P* < 0.05 was considered statistically significant. All statistical analyses were performed using MATLAB or GraphPad Prism 8 software.

## Results

### Oncology drug screen reveals proteasome inhibitor sensitivity in HCC PDXOs

**Fig. 1:**
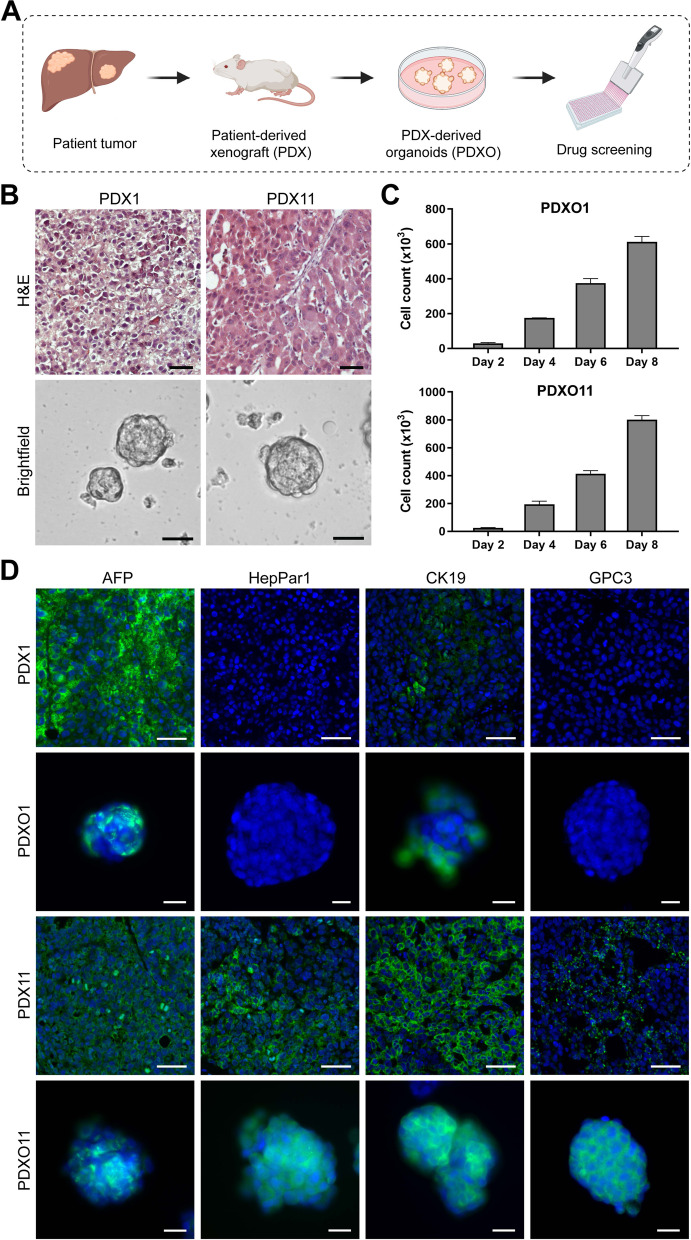
Establishment of HCC PDXO lines from PDX tumors. **A** Schematic diagram illustrating the generation of HCC PDXs and PDXOs from human patient HCC tumors for subsequent drug screening assays. **B** H&E and brightfield images of PDX1 and PDX11 tumors and their corresponding PDX-derived organoids. Scale bar = 50 μm. **C** Mean viable cell count of HCC PDXOs grown in culture, measured via trypan blue exclusion assay at Days 2, 4, 6 and 8. Data shown as mean ± SD, *n* = 3. **D** Immunofluorescence staining of HCC markers (AFP, HepPar1, CK19, GPC3) in HCC PDX1 and 11 and their corresponding matched PDXOs. DAPI nuclear stain is shown in blue. Scale bars = 50 μm (PDX), 20 μm (PDXO)

To identify potential candidate drugs that are effective against HCC tumor cells, we performed an oncology drug screen on a panel of HCC PDXO lines derived from their respective PDX tumors (Fig. 1A). HCC PDXOs grown in culture were viable and maintained proliferative capacity up to 8 days (Fig. 1B and C). These PDXOs also shared similar profiles of HCC markers such as alpha-fetoprotein (AFP), hepatocyte paraffin 1 (HepPar1), cytokeratin 19 (CK19) and glypican 3 (GPC3) as that of the parental PDXs (Fig. 1D).

**Fig. 2:**
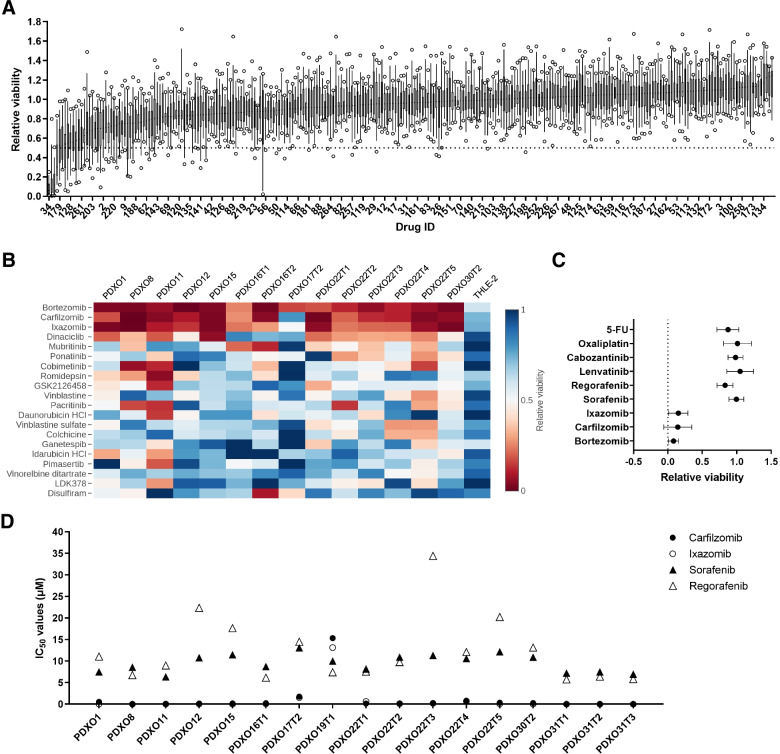
Drug screen highlights proteasome inhibitors as top-ranking effective drugs against HCC PDXOs. **A** Median cell viability of 268 drugs (treated at 1 μM) across 14 PDXO lines displayed in a box-and-whisker plot along with the variance. Dotted line indicates 50% viability. **B** Heatmap showing relative viability of top 20 ranked drugs for each HCC PDXO line and a non-tumorigenic liver epithelial cell control, THLE-2. Color-coded values of the heatmap represent the mean of 2 technical replicates. **C** Mean cell killing effects of proteasome inhibitors (bortezomib, carfilzomib, ixazomib) and common HCC clinical drugs (sorafenib, regorafenib, lenvatinib, cabozantinib, oxaliplatin, 5-FU) in 14 PDXO lines as derived from drug screen at 1 μM. Data presented as mean ± SD, *n* = 14. *P* < 0.001 comparing each proteasome inhibitor with other drugs (Kruskal-Wallis test). **D** IC_50_ values of carfilzomib, ixazomib, sorafenib, and regorafenib in HCC PDXO lines

For the initial drug screen, a total of 268 compounds from an oncology drug library, which includes 165 FDA-approved compounds, were administered at 1 μM concentration on 14 PDXO lines. Only four drugs—bortezomib, carfilzomib, ixazomib, and dinaciclib—reduced median cell viability by more than 50% (Fig. 2A and Table S2). The top three drugs bortezomib, carfilzomib and ixazomib are proteasome inhibitors, while dinaciclib is an inhibitor of cyclin-dependent kinases (CDKs) 1, 2, 5 and 9 [45]. When we examined the response of individual PDXO lines towards these drugs by using a 50% relative viability cut-off as an indicator of tumor cell killing efficacy, bortezomib was pan-effective across the different PDXOs, whereas carfilzomib, ixazomib, and dinaciclib had at least one PDXO line with suboptimal response (Fig. 2B). Nevertheless, with the exception of the more drug resistant line PDXO17T2, both carfilzomib and ixazomib were able to induce over 70% cell death in all other PDXO lines tested, highlighting the efficacy of proteasome inhibitors against HCC tumor cells.

A non-tumorigenic liver epithelial cell line, THLE-2, was also included in the drug screen to examine potential off-target toxicities of these drugs. Among the three proteasome inhibitors, bortezomib had the most effect in reducing THLE-2 cell viability (Fig. 2B), suggesting greater potential toxicity on non-tumorigenic cells. In fact, this observation mimics the poorer safety profiles of bortezomib as compared to the second-generation proteasome inhibitors carfilzomib and ixazomib reported in clinical trials [46, 47].

The efficacy of proteasome inhibitors was also compared against drugs approved for clinical use in HCC (sorafenib, regorafenib, lenvatinib, and cabozantinib) and two common chemotherapy drugs that have been used in HCC treatment (oxaliplatin and 5-fluorouracil) [48–50]. The proteasome inhibitors showed significantly greater cytotoxic effects on HCC PDXOs than the clinical and chemotherapy drugs when administered at 1 μM concentration (*P* < 0.0001, Kruskal-Wallis test) (Fig. 2C). The enhanced sensitivity of HCC PDXOs towards proteasome inhibitors was further validated in a separate experiment, in which carfilzomib and ixazomib exhibited lower IC_50_ values than the clinical HCC drugs (sorafenib and regorafenib) in various PDXO lines, except for PDXO19T1 (Fig. 2D). Collectively, these results indicate that HCC PDXOs are generally susceptible to proteasome inhibitors.

### Proteasome inhibitors demonstrate in vitro efficacy against HCC PDXOs

**Fig. 3:**
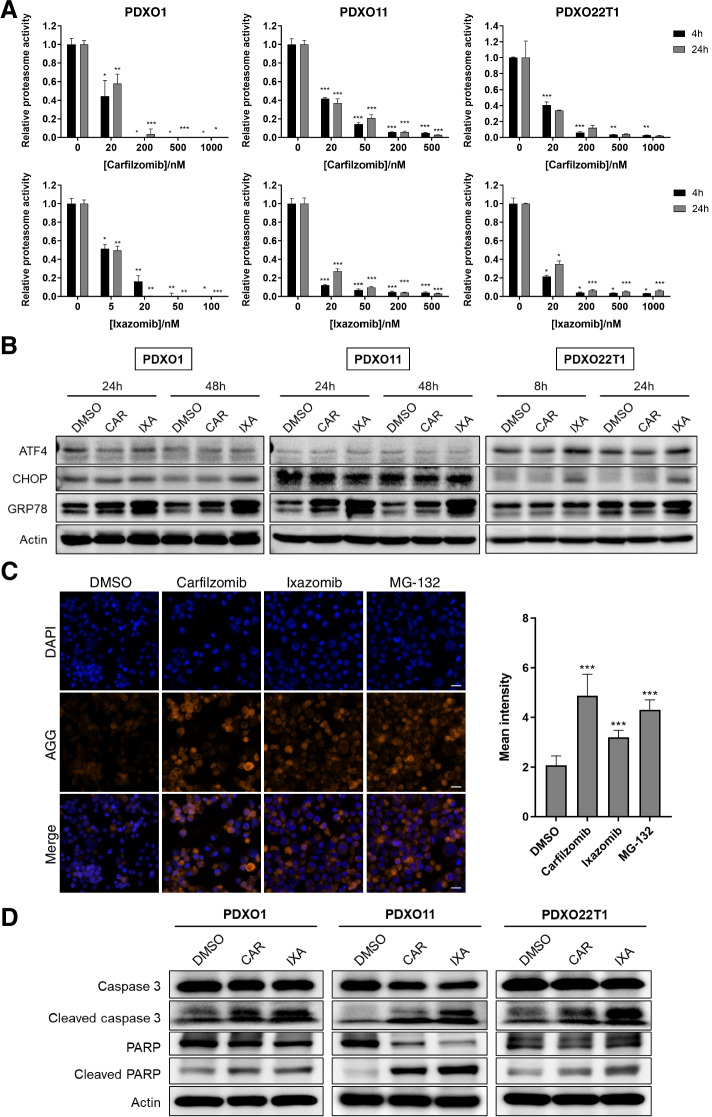
Proteasome inhibitors induce anti-tumor effects in HCC PDXOs. **A** Proteasome activity in HCC PDXO1, 11 and 22 T1 following treatment with carfilzomib and ixazomib for 4 and 24 hours. Data presented as mean ± SD, *n* = 3. *, *P* < 0.05; **, *P* < 0.01; ***, *P* < 0.001, compared to respective time-point controls (0 nM). Statistical significance was determined by two-tailed Student’s *t* test. **B** Immunoblot analysis of ER stress pathway proteins upon treatment with carfilzomib (CAR) and ixazomib (IXA) in HCC PDXOs. **C** Immunofluorescence staining of aggresome (AGG) formation in PDX11 organoids with corresponding quantification of aggresome expression. MG-132 was used as a positive control. Scale bar = 20 μm. Data presented as mean ± SD, *n* = 10. **D** Immunoblotting of markers of apoptosis in HCC PDXOs treated with carfilzomib (CAR) and ixazomib (IXA)

Given bortezomib’s higher cytotoxic effects on THLE-2 cells (Fig. 2B), we decided to focus on carfilzomib and ixazomib for subsequent experiments and efficacy studies. The potency of carfilzomib and ixazomib in specifically inhibiting proteasome activity was examined by measuring the chymotrypsin-like activity of proteasomal β5 subunit following treatment with the inhibitors. Within 4 hours of carfilzomib or ixazomib exposure at concentrations below their respective IC_50_ values, proteasome activity was decreased by more than 50% in PDXOs 1, 11 and 22 T1, and was sustained for up to 24 hours (Fig. 3A).

Next, we assessed the functional effects of proteasome inhibition in HCC PDXOs by looking at the endoplasmic reticulum (ER) stress pathway. Studies have shown that the disruption of elevated proteasome activity in rapidly dividing cancer cells can result in ER stress and the subsequent activation of the unfolded protein response (UPR), which if prolonged can lead to stress-related apoptosis [51–54]. Hence, we examined the effects of proteasome inhibitor treatment on the expression of several proteins known to be regulated by the UPR. PDXOs 1, 11 and 22 T1 treated with carfilzomib and ixazomib for 24 hours displayed upregulation of the molecular chaperone, glucose-regulated protein 78 kDa (GRP78), and activating transcription factor 4 (ATF4), which mediates the transcription of adaptive genes in response to cellular stress, including pro-apoptotic C/EBP homologous protein (CHOP) (Fig. 3B) [55]. Interestingly, the changes in expression of UPR proteins following proteasome inhibition varied slightly among the PDXO lines tested, and could imply differences in duration or mechanistic response to ER stress in these cells.

We also measured the formation of aggresomes, a known hallmark of proteasome inhibition that arises from aggregates of misfolded or unfolded proteins [56–58]. As proteasome inhibition impedes protein degradation by the ubiquitin-proteasome system, the resulting accumulation of such proteins activates the aggresome-autophagy pathway as a compensatory clearing mechanism [56]. Indeed, treatment with carfilzomib and ixazomib for 24 hours significantly increased aggresome formation in HCC PDXO1 cells (Fig. 3C).

More importantly, the pro-apoptotic effects of carfilzomib and ixazomib were demonstrated by the increase in population of positively stained Annexin V/PI cells after treatment via flow cytometric analysis (Fig. S1). Carfilzomib and ixazomib treatment also induced cleavage of apoptotic markers caspase 3 and its downstream target poly (ADP-ribose) polymerase (PARP) in HCC PDXOs (Fig. 3D). These results highlight the efficacy of proteasome inhibitors against HCC tumor cells and their potential application for HCC therapy.

### QPOP identifies the combination of ixazomib and dinaciclib (Ixa + Dina) to be effective against HCC PDXOs

To rationally design and identify optimal proteasome inhibitor-based drug combinations, we conducted QPOP analysis using 155 experimental test combinations (Table S3) derived from an orthogonal array composite design (OACD) dataset of nine drugs at three different dosages (Table S4). The nine-drug set consisted of three top-ranked drug candidates from the oncology drug screen (ixazomib, carfilzomib, dinaciclib) and six clinically relevant HCC drugs (sorafenib, regorafenib, lenvatinib, cabozantinib, oxaliplatin and 5-FU). QPOP was carried out on 18 HCC PDXO lines and a control cell line, THLE-2, to identify drug combinations with maximal tumor cell killing and minimal toxic effects on non-tumorigenic cells. The difference in normalized cell viability between PDXO and THLE-2 (viability_THLE2_ – viability_PDXO_) was used as input data points for response surface mapping (second order regression), and all possible two-drug combinations were then ranked based on the predicted therapeutic output.

**Fig. 4:**
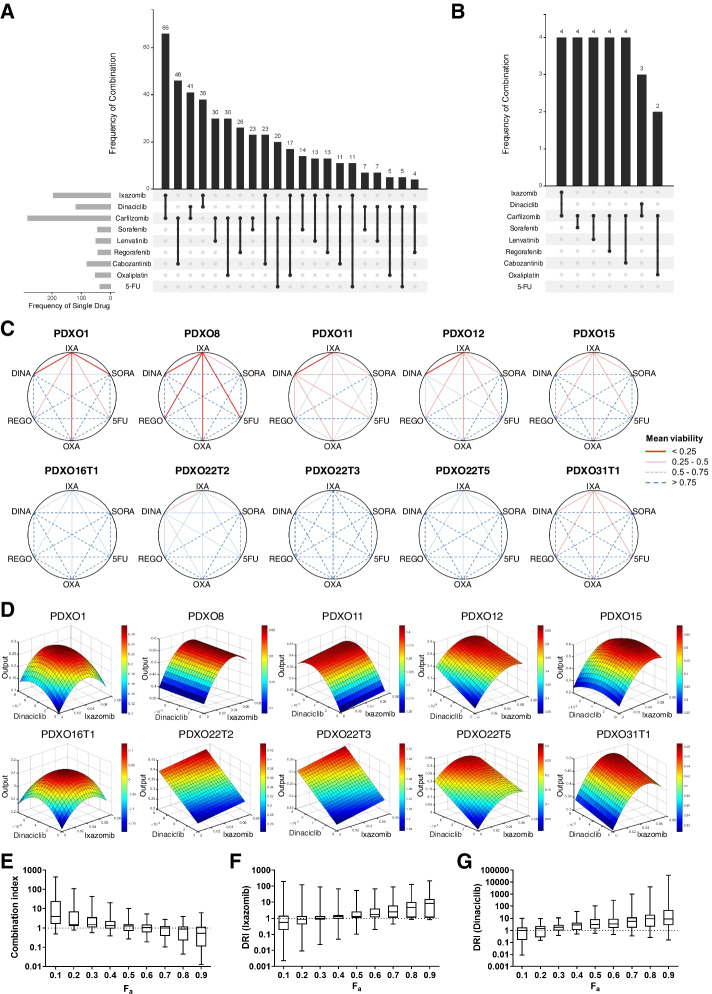
QPOP identifies Ixazomib and Dinaciclib (Ixa + Dina) as an effective drug combination against HCC PDXOs. **A** Frequency of individual drugs and combinations appearing within the top 25 ranked two-drug combinations in all PDXO lines tested. **B** Frequency of drug combinations appearing within the top 25 ranked two-drug combinations in THLE-2. **C** Polygonograms depicting QPOP-derived two-drug interaction effects on PDXO viability between ixazomib (IXA), dinaciclib (DINA), sorafenib (SORA), regorafenib (REGO), oxaliplatin (OXA), and 5-fluorouracil (5FU) in representative HCC PDXOs. **D** Parabolic response surface maps showing the effects of ixazomib and dinaciclib on the therapeutic output (viability_THLE2_ – viability_PDXO_) in representative PDXOs. **E** Median and range of combination index values for Ixa + Dina in 17 PDXO lines. F_a_, fraction of cells affected. **F** and **G** Median and range of dose reduction index (DRI) values for ixazomib (**F**) and dinaciclib (**G**) across 17 PDXO lines

While HCC PDXOs demonstrated differential sensitivities to each drug combination (Fig. S2A and Table S5), we sought to identify proteasome inhibitor-based drug combinations that were commonly effective across the HCC PDXO lines tested. Based on the QPOP-derived output of PDXO cell viability, all two-drug combinations were ranked in terms of efficacy, and the frequencies at which each drug and combination appeared in the top 25 ranks across all 18 PDXO lines were determined (Fig. 4A). Proteasome inhibitors carfilzomib and ixazomib were the two most frequently occurring drugs in the top-ranking combinations, while the four most common drug combinations among the PDXOs included ixazomib with carfilzomib or dinaciclib, and carfilzomib with dinaciclib or cabozantinib (Fig. 4A). However, when we ranked the drug combinations in terms of their effects on THLE-2 cell viability, carfilzomib-based combinations appeared most often in the top ranks (Fig. 4B), suggesting more potent effects of these drug combinations on normal cells. Hence, given the potential toxicities associated with carfilzomib-based drug combinations, we identified the combination of ixazomib and dinaciclib (Ixa + Dina) as the optimal top-ranking proteasome inhibitor-based drug combination against HCC PDXOs (Fig. 4A). This was further demonstrated when we compared the QPOP-predicted output values of Ixa + Dina against other ixazomib-based combinations within each PDXO (Fig. 4C). Ixa + Dina showed greater decrease in tumor cell viability as compared to combinations of ixazomib with other drugs and other two-drug combinations (Fig. 4C and Fig. S2B).

In addition, the parabolic response surface maps generated from QPOP analysis indicated a synergistic interaction between ixazomib and dinaciclib, which was depicted by the increase in therapeutic output (viability_THLE2_ – viability_PDXO_) with increasing drug concentrations (Fig. 4D and Fig. S2C). To validate this interaction, we performed single-drug and combination dose-response assays on the HCC PDXOs to obtain respective IC_50_ values for combination index (CI) and dose reduction index (DRI) calculations based on the Chou-Talalay method [43]. Briefly, CI values denote the type of interaction between drugs in combination, which can be synergistic (CI < 1), additive (CI = 1), or antagonistic (CI > 1). Conversely, DRI defines the amount of dose reduction allowed for a drug when used in combination to achieve the same cell killing effect as when used alone and is categorized into favorable (DRI > 1), unfavorable (DRI < 1), or no dose reduction (DRI = 1). Both CI and DRI values can change depending on the concentration of drugs used, as represented by the fraction of cells affected or killed (F_a_) [43]. For the combination of ixazomib and dinaciclib, the interaction between both drugs showed an increasingly synergistic trend (CI < 1) with increasing drug concentrations across the panel of HCC PDXO lines tested (Fig. 4E). This was supported by progressively favorable dose reductions (DRI > 1) for both ixazomib (Fig. 4F) and dinaciclib (Fig. 4G) when used in combination. These results indicate that when administered concurrently, the combination of Ixa + Dina can contribute to a synergistic increase in efficacy against HCC tumor cells, while allowing for reductions in dosages of both drugs to minimize potential toxic side effects.

### Ixa + Dina combination induces anti-proliferative and pro-apoptotic effects in HCC PDXOs

**Fig. 5:**
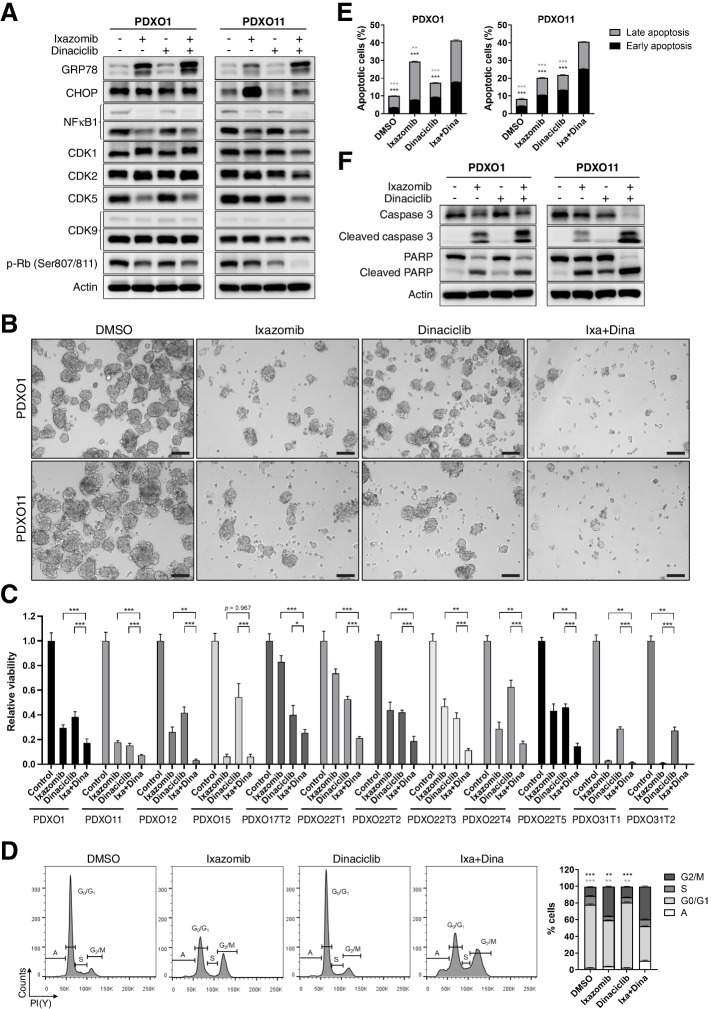
Ixa + Dina combination demonstrates increased in vitro efficacy against HCC PDXOs. **A** Immunoblotting of ER stress, NFκB and cell cycle proteins in representative PDXO lines, PDXO1 and 11. **B** Brightfield images of HCC PDXOs treated with vehicle (DMSO), ixazomib, dinaciclib or Ixa + Dina. Scale bar = 100 μm. **C** Average viability of PDXOs when treated with ixazomib (0.1 μM) and dinaciclib (0.01 μM) as monotherapy and in combination. Data presented as mean ± SD, *n* = 5. *, *P* < 0.05; **, *P* < 0.01; ***, *P* < 0.001. **D** Histograms of cell cycle analysis and the corresponding quantification of the proportion of cells in A (apoptotic), G0/G1, S, and G2/M phases of the cell cycle after 24 h drug treatment in PDXO11. Data shown as mean ± SD, *n* = 3. *, *P* < 0.05; **, *P* < 0.01; ***, *P* < 0.001, compared to Ixa + Dina. Black and grey asterisks indicate comparison for G2/M and apoptotic phases, respectively. **E** Quantification of annexin V/PI-stained PDXO1 and PDXO11 after Ixa + Dina treatment for 24 hours. Data presented as mean ± SD, *n* = 3. **, *P* < 0.01; ***, *P* < 0.001, compared to Ixa + Dina. Black and grey asterisks indicate comparison for early and late apoptosis groups, respectively. **F** Immunoblotting of apoptotic markers after treatment with Ixa + Dina in HCC PDXOs. All statistical analyses were performed using two-tailed Student’s *t* test

Before investigating the anti-tumor effects of Ixa + Dina combination in HCC PDXOs, we first validated the activity of each drug by examining their known molecular effects via immunoblot analysis. As ixazomib has been reported to induce the UPR and repress NFκB signaling in multiple cancers [25, 59, 60], the expression of several ER stress pathway proteins and NFκB1 were used as surrogate markers of ixazomib activity. On the other hand, CDK-specific phosphorylation of Rb protein was used as a mechanism-based marker of dinaciclib activity [45, 61]. HCC PDX1 and PDX11 organoids treated with ixazomib or dinaciclib at their respective combination IC_50_ values demonstrated increased levels of ER stress-induced protein GRP78, reduced NFκB1 expression, and decreased phosphorylation of Rb (p-Rb) (Fig. 5A). Interestingly, simultaneous administration of both drugs in combination further augmented the changes in expression of several proteins such as GRP78, p-Rb, and CDK5 in both PDXO lines, suggesting possible common molecular effects between ixazomib and dinaciclib.

We next assessed the efficacy of Ixa + Dina combination by exploring its effects on tumor cell proliferation, survival, and apoptosis. Concurrent administration of ixazomib and dinaciclib showed greater decrease in both number and size of PDX1 and PDX11 organoids as compared to either drug alone (Fig. 5B). The inhibitory effects of Ixa + Dina on tumor cell survival was also demonstrated by the greater reduction in PDXO cell viability in multiple PDXO lines (Fig. 5C). In addition, cell cycle analysis of combination-treated PDXOs after 24 hours revealed increased accumulation of cells in the G2/M phase when compared to single-drug and control-treated cells (Fig. 5D). The results also showed a larger proportion of cells in the apoptotic phase following Ixa + Dina treatment (Fig. 5D). The induction of apoptotic cell death was further confirmed by the substantial increase in Annexin V/PI-positive stained cells (Fig. 5E and Fig. S3) and upregulation of cleaved caspase 3 and cleaved PARP protein levels in PDXOs treated with Ixa + Dina combination (Fig. 5F). Taken together, our findings demonstrate that the combination of Ixa + Dina suppresses tumor cell proliferation and promotes apoptotic cell death in HCC PDXOs to a greater extent than each drug alone, providing further evidence for the synergistic interaction of both drugs in promoting tumor inhibitory effects.

### JNK signaling mediates pro-apoptotic effects of Ixa + Dina in HCC PDXOs

**Fig. 6:**
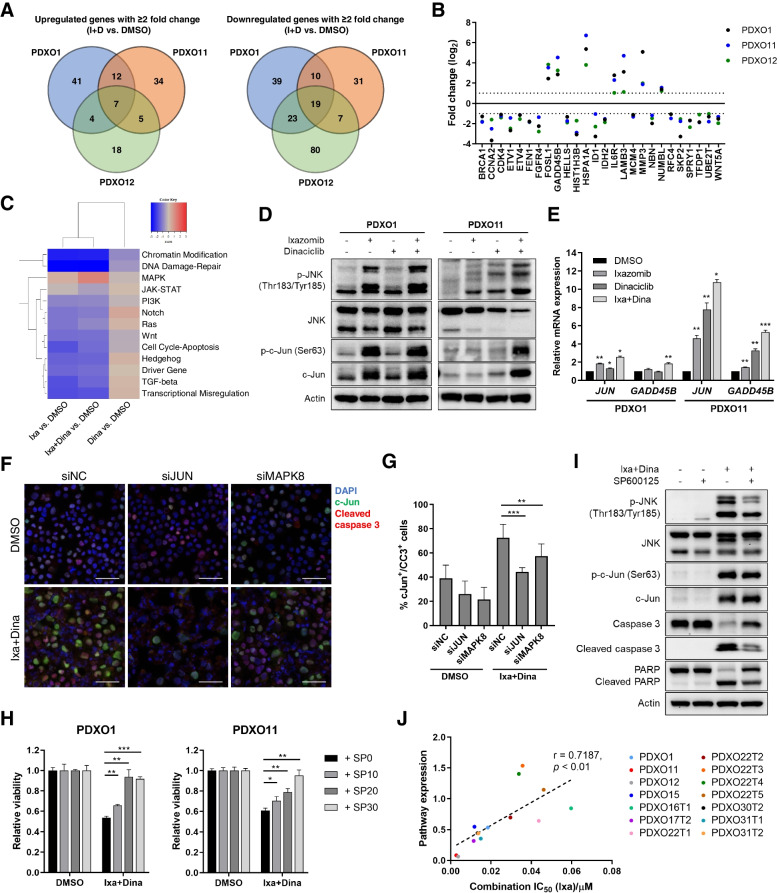
Ixa + Dina induced apoptosis is mediated by upregulated JNK signaling. **A** Venn diagram showing overlapping genes with at least 2-fold change upregulation or downregulation after treatment with Ixa + Dina versus DMSO control in PDXO1, 11 and 12. **B** Log_2_ fold changes of common differentially expressed genes between Ixa + Dina and DMSO groups for PDXO1, 11, and 12. Dotted lines indicate cut-off at fold change of 2. **C** Heatmap of directed global significance scores based on NanoString gene set analysis. **D** and **E** Immunoblot of JNK pathway proteins (**D**) and quantitative RT-PCR analysis of c-Jun transcriptional activity (**E**) after treatment with ixazomib and dinaciclib in HCC PDXO1 and 11. Data presented as mean ± SD, *n* = 3. *, *P* < 0.05; **, *P* < 0.01; ***, *P* < 0.001, compared to respective DMSO controls. **F** Immunofluorescence co-staining of c-Jun and cleaved caspase 3 in Ixa + Dina treated HCC PDXO1 in the presence of siRNAs targeting c-Jun (siJUN) and JNK (siMAPK8). Green fluorescence indicates c-Jun expression; Red indicates cleaved caspase 3; Blue indicates DAPI staining. Scale bar = 50 μm. **G** Quantification of tumor cells that co-express both c-Jun and cleaved caspase 3 (cJun^+^/CC3^+^) per field. Data presented as mean ± SD, *n* = 10. **, *P* < 0.01; ***, *P* < 0.001. **H** Relative viability of Ixa + Dina treated PDXO1 and PDXO11 in the presence of a JNK inhibitor (SP600125) at 10 μM (SP10), 20 μM (SP20), and 30 μM (SP30) concentrations. Data presented as mean ± SD, *n* = 3. *, *P* < 0.05; **, *P* < 0.01; ***, *P* < 0.001. All statistical analyses were performed using two-tailed Student’s *t* test. **I** Immunoblotting of apoptotic markers after Ixa + Dina and JNK inhibitor treatment in HCC PDXO11. **J** Correlation (Pearson’s r) between basal JNK pathway expression and Ixa + Dina sensitivity (represented by combination IC_50_ values of ixazomib) in HCC PDXOs. JNK pathway expression was determined using the sum of p-JNK/JNK and c-Jun protein expression quantified from immunoblot analysis

To interrogate the molecular pathways and genes affected by the combination of Ixa + Dina in HCC, we conducted transcriptomic analyses of 730 cancer-related genes (NanoString PanCancer Pathways panel) in three representative PDXO lines (PDXO1, 11, 12) following treatment with ixazomib and dinaciclib. When we compared genes that showed at least two-fold-change difference after Ixa + Dina treatment, a total of 26 genes were found to be differentially expressed in all the PDXO lines tested (Fig. 6A). These include 7 upregulated genes *FOSL1*, *GADD45B*, *HSPA1A*, *IL6R*, *LAMB3*, *MMP3*, *NUMBL*, and 19 downregulated genes *BRCA1*, *CCNA2*, *CDK4*, *ETV1*, *ETV4*, *FEN1*, *FGFR4*, *HELLS*, *HIST1H3B*, *ID1*, *IDH2*, *MCM4*, *NBN*, *RFC4*, *SKP2*, *SPRY1*, *TFDP1*, *UBE2T*, *WNT5A* (Fig. 6B).

To obtain an overview of potential pathway alterations induced by Ixa + Dina, we also performed a NanoString gene set analysis, which summarizes the overall changes in gene expression within 13 defined gene sets using directed global significance scores. These scores were calculated based on t-statistics from differential expression analysis across the three PDXO lines and indicate the tendency of gene sets or pathways to have over- or under-expressed genes. Interestingly, while most of the gene sets exhibited decreased pathway scores following drug exposure, the combination of Ixa + Dina showed a greater increase in MAPK pathway scores as compared to single-drug or control-treated groups (Fig. 6C). This suggested a potential role of MAPK pathway in mediating the combined effects of Ixa + Dina in HCC tumor cells.

Moving forward, to determine which MAPK pathway was affected by Ixa + Dina, we examined the effects of the drug combination in relation to the three main MAPKs—JNK, p38 and ERK. Notably, JNK was consistently phosphorylated in both PDXO1 and PDXO11 in response to Ixa + Dina treatment, but p38 and ERK showed varying trends between both PDXOs (Fig. 6D and Fig. S4A). The activation of JNK signaling by Ixa + Dina was also seen in the increase in phosphorylation of its downstream target c-Jun (Fig. 6D), which further promoted transcription of its encoding gene (*JUN*) and other targets such as growth arrest and DNA damage-inducible beta (*GADD45B*) [62] (Fig. 6E). In fact, *GADD45B* was among the genes that were significantly upregulated in all three HCC PDXO lines used for transcriptomic analysis (Fig. 6B). This suggests that JNK signaling may play a more dominant role in mediating the tumor suppressive effects of Ixa + Dina combination in HCC PDXOs.

As JNK signaling has been reported to activate cellular apoptosis [63–65], we sought to investigate the relationship between JNK signaling and Ixa + Dina induced tumor cell death in HCC PDXOs. Multiplex immunofluorescence staining assays demonstrated a 33% increase in the proportion of tumor cells expressing both c-Jun and cleaved caspase 3 (cJun^+^/CC3^+^) after treatment with Ixa + Dina (Fig. 6F and G). Notably, this trend was diminished upon abrogation of JNK signaling via siRNA-mediated knockdown of JNK (siMAPK8) or c-Jun (siJUN) (Fig. 6F and G), which were validated by qRT-PCR and immunoblot analysis (Fig. S4B and C). In fact, siRNA-mediated inhibition of c-Jun in Ixa + Dina treated PDXOs significantly reduced the proportion of cJun^+^/CC3^+^ cells compared to control (I + D + siNC) levels (Fig. 6G). These results were further corroborated by chemical loss-of-function studies using a small molecule inhibitor of JNK, SP600125. Pre-treatment with SP600125 diminished the drug combination-induced reduction in HCC PDXO viability in a dose-dependent manner (Fig. 6H). Administration of SP600125 also decreased the cleavage of caspase 3 and PARP induced by Ixa + Dina treatment in the tumor cells (Fig. 6I), indicating that inhibition of JNK activity can confer protection from drug combination-mediated apoptosis.

Since upregulation of JNK signaling is a key mediator of Ixa + Dina’s anti-tumor effects in tested HCC PDXOs, we hypothesized that basal activity levels of the pathway could affect the tumor cell sensitivity to the drug combination. Hence, we sought to determine if basal JNK pathway activity was associated with susceptibility of HCC PDXOs to Ixa + Dina treatment. Immunoblot analysis of basal JNK signaling pathway demonstrated a range of expression levels among the various PDXO lines (Fig. S4D). Using a pathway expression score derived from the sum of p-JNK/JNK and c-Jun expression levels (normalized to THLE-2), we examined the correlation of the pathway expression score with the combination IC_50_ values of ixazomib, which was used as an indicator of drug response sensitivity. Interestingly, with the exception of PDXO8, we observed a significant positive correlation (*r* = 0.7187, *P* < 0.01) between JNK pathway score and drug combination IC_50_ values (Fig. 6J), indicating that HCC tumor cells with lower basal levels of JNK signaling were more sensitive to Ixa + Dina treatment. This is further evidenced by PDXO1 and PDXO11 being in the bottom 50th percentile in basal JNK expression (Fig. 6J).

Collectively, these findings highlight the role of JNK signaling pathway in mediating the pro-apoptotic effects of Ixa + Dina combination in HCC tumor cells. Furthermore, basal JNK signaling activity may serve as a predictive biomarker of drug sensitivity and response to Ixa + Dina in HCC.

### Ixa + Dina combination inhibits HCC PDX tumor progression in vivo

**Fig. 7:**
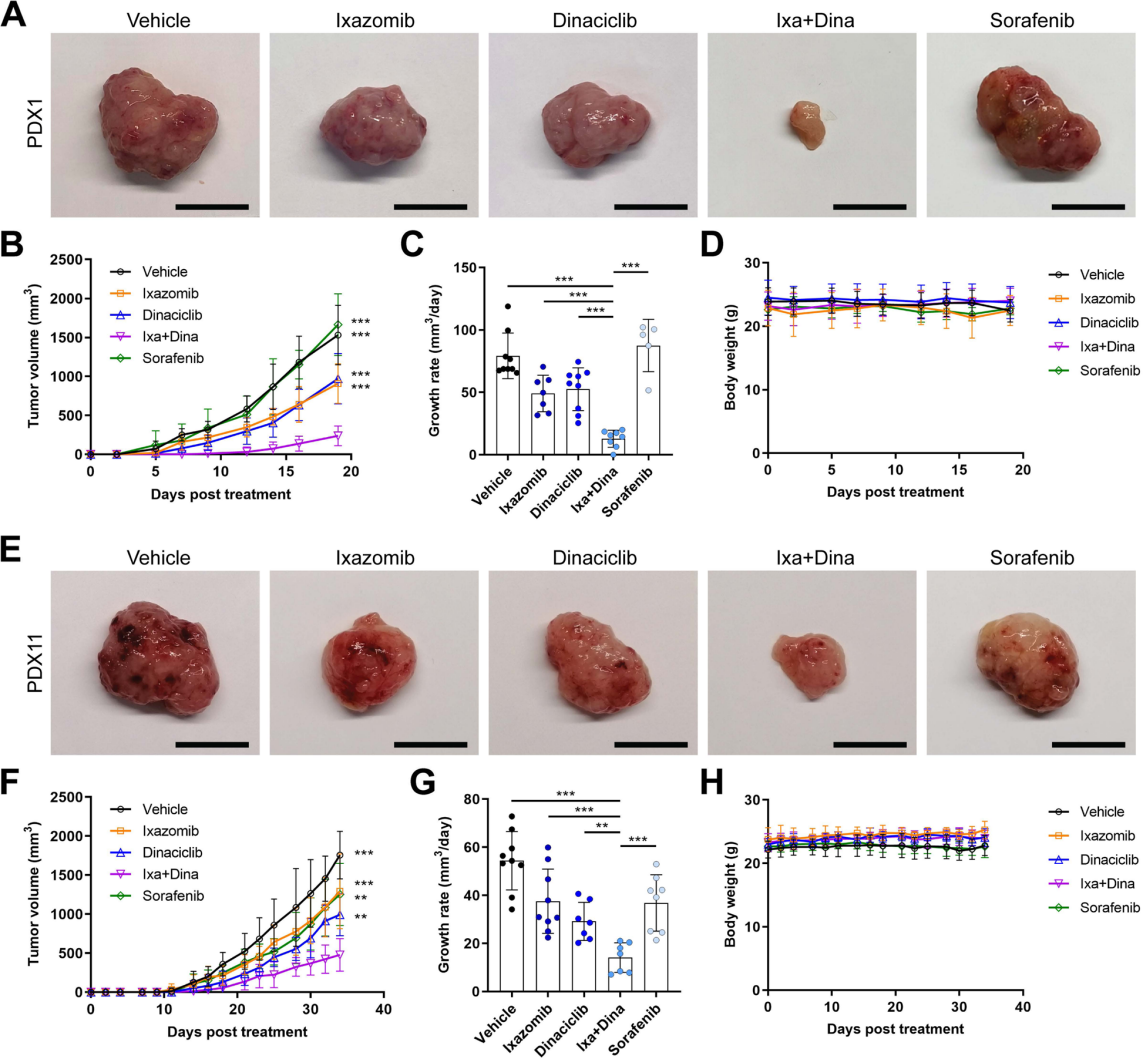
Ixa + Dina combination impairs tumor growth in HCC PDXs. **A** Representative gross tumor images of HCC PDX1 after treatment with vehicle, ixazomib (7 mg/kg), dinaciclib (5 mg/kg), Ixa + Dina, and sorafenib (30 mg/kg). Scale bar = 1 cm. **B** and **C** Tumor volumes (**B**) and growth rates (**C**) of PDX1 tumors after drug treatment. Data presented as mean ± SD, *n* = 5 to 9. ***, *P* < 0.001 compared to Ixa + Dina group. **D** Body weight changes of mice bearing HCC PDX1 tumors over the course of drug treatment. Data presented as mean ± SD, *n* = 5 to 9. **E** Representative gross tumor images of HCC PDX11 after treatment with vehicle, ixazomib (7 mg/kg), dinaciclib (5 mg/kg), Ixa + Dina, and sorafenib (30 mg/kg). Scale bar = 1 cm. **F** and **G** PDX11 tumor volumes (**F**) and growth rates (**G**) after drug treatments. Data presented as mean ± SD, *n* = 7 to 9. **, *P* < 0.01; ***, *P* < 0.001 compared to Ixa + Dina group. **H** Mean body weight of PDX11 tumor-bearing mice during the course of drug treatment. Data presented as mean ± SD, *n* = 7 to 9. All statistical analyses were performed using two-tailed Student’s *t* test

To evaluate the tumor suppressive effects of Ixa + Dina combination in vivo, we treated mice bearing HCC PDX1 and PDX11 tumors with drugs as monotherapy and in combination 1 week after tumor implantation. Tumor volumes were measured to determine tumor growth rates, and mice were euthanized once tumors in the vehicle-treated group reached endpoint volume. PDX1 tumor-bearing mice treated with Ixa + Dina combination developed significantly smaller tumors (Fig. 7A and B) and had the lowest growth rate as compared to vehicle- and single-drug-treated mice (Fig. 7C; *P* < 0.001). This trend was also observed in PDX11 tumors, in which administration of Ixa + Dina produced a more robust inhibition of tumor progression than the single-drug treatments (Fig. 7E-G; *P* < 0.01, Ixa + Dina vs. Dinaciclib; *P* < 0.001, Ixa + Dina vs. vehicle and Ixazomib), despite PDX11 tumors developing slower in vivo as compared to PDX1 tumors. More importantly, we demonstrated that Ixa + Dina was more effective than the standard-of-care regimen sorafenib at impairing tumor progression in both HCC PDX lines (Fig. 7A-C, E-G).

Potential therapy-related toxicities were also examined by measuring changes in the body weight of the animals following drug administration. No significant loss of body weight was observed during and after the course of drug treatment in both PDX1 and PDX11 tumor-bearing mice (Fig. 7D and H), suggesting that the drug dosages administered were tolerable. In addition, the combination of ixazomib and dinaciclib showed no observable toxic side effects based on histological analysis of the livers, kidneys, and spleens collected from drug-treated mice (Fig. S5).

**Fig. 8:**
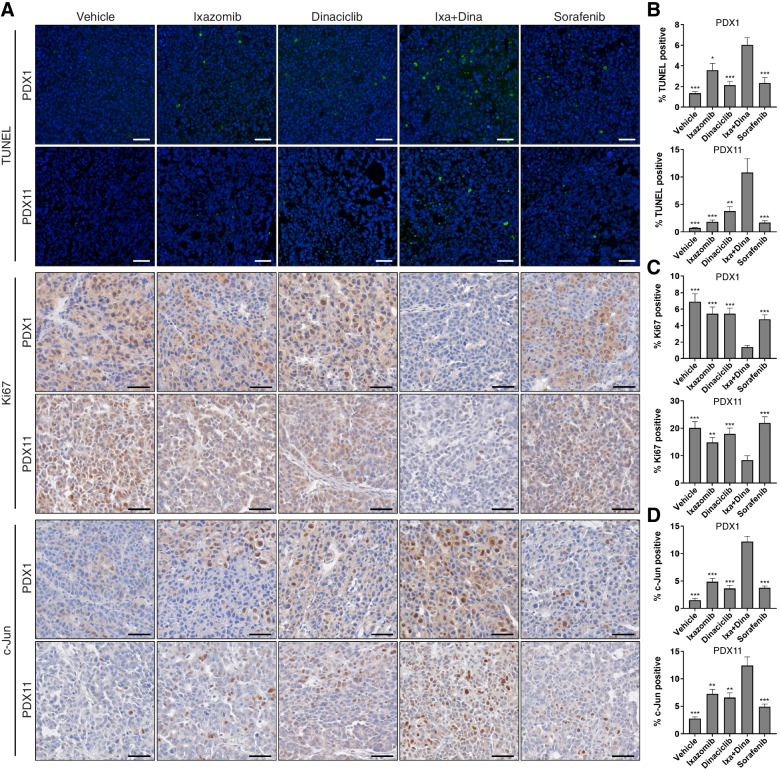
Ixa + Dina treatment exhibits anti-tumor effects in vivo. **A** Representative images of TUNEL, Ki67, and c-Jun immunohistochemical staining in PDX1 and PDX11 tumors after drug treatment. Scale bar = 50 μm. **B** - **D** Corresponding quantification of TUNEL (**B**), Ki67 (**C**), and c-Jun (**D**) expression in HCC PDX tumors. Data presented as mean ± SEM, *n* ≥ 80 fields. **, *P* < 0.01; ***, *P* < 0.001 compared to Ixa + Dina group. All statistical analyses were performed using two-tailed Student’s *t* test

The increased in vivo efficacy of ixazomib and dinaciclib in combination was further demonstrated by the significantly higher levels of apoptosis in Ixa + Dina treated tumors as compared to ixazomib (*P* < 0.05), dinaciclib (*P* < 0.01), or sorafenib (*P* < 0.001) treatment groups, as shown via terminal deoxynucleotidyl transferase-mediated dUTP nick-end labeling (TUNEL) analysis (Fig. 8A and B). Furthermore, immunohistochemical analysis of PDX1 and PDX11 tumor tissues also revealed a significant reduction in Ki67 protein levels (Fig. 8A and C; *P* < 0.01) and concurrent increase in c-Jun levels (Fig. 8A and D; *P* < 0.01) after Ixa + Dina combination treatment, as compared to vehicle, single-drug, and sorafenib treated tumors. The increased apoptotic response and decreased proliferative potential in PDX tumors following treatment with Ixa + Dina are consistent with the results observed in HCC PDXOs. Taken together, our findings demonstrate that Ixa + Dina is a tolerable combination regimen that can more effectively impair HCC tumor progression in vivo than monotherapy of either drug or even the standard-of-care sorafenib.

## Discussion

The need for more effective therapeutic options for advanced HCC has fueled research efforts towards developing various treatment modalities and potential drug repurposing strategies. Proteasome inhibitors have achieved much success in getting clinical approval for the treatment of hematological malignancies and have thus been tested in clinical trials for several solid tumors as well, including cancers of the head and neck, lung, and colon [21–23]. While early trials of first-in-class proteasome inhibitor bortezomib for HCC showed no clinical benefit or advantage over the standard first-line regimen, sorafenib [23], this study provides evidence that the right proteasome inhibitor can be useful in treating HCC when applied in combination with the appropriate drugs.

The proper design of effective combinatorial treatments, however, remains a challenging task. The unsuccessful translation of numerous drug combinations [27–29] could be attributed to poor drug combination design, disease heterogeneity and a lack of understanding of critical mechanisms or drivers of tumor progression in HCC [30, 66]. For molecularly targeted therapies, including proteasome inhibitors, identifying an effective combination is even more difficult given the large drug search space and limited drug-drug interactions that may exist amongst targeted therapies. Efficient analysis of quantitative phenotypic datasets by QPOP following OACD drug combination treatments could overcome these hurdles, particularly when paired with patient-derived avatars. QPOP has been primarily used to identify effective drug combinations for hematological malignancies [31, 67] and could accurately predict combinations that resulted in clinical response in refractory T-cell lymphoma patients [36]. Given the initial clinical translation success observed in hematological malignancies, QPOP applied to a set of HCC PDXOs should provide clinically promising combinations of drugs that can interact with proteasome inhibitors to effectively inhibit HCC progression.

Thus, through QPOP, we identified Ixa + Dina as one of the most frequently reoccurring top-ranked proteasome inhibitor-based drug combination across our panel of HCC PDXOs. Although our drug set included both carfilzomib and ixazomib, QPOP was able to prioritize an ixazomib-specific drug combination for subsequent studies. Given the ability of QPOP to rank all possible drug combinations and compare the combinatorial therapy potential of drugs within the same class, this approach may be useful for prioritizing molecular targeted therapies for further clinical development where multiple promising drugs within a single class exist. Through mapping all drug-drug interactions within the drug set from a single OACD experimental test dataset, QPOP predicted synergism between ixazomib and dinaciclib. Subsequent in vitro and in vivo validation studies confirmed that ixazomib synergized with dinaciclib, resulting in enhanced tumor suppressive effects and better efficacy than sorafenib against our HCC PDX and PDXOs.

In multiple myeloma, the combination of dinaciclib and proteasome inhibitor bortezomib has been reported be effective against tumor cells [68], but this has not been explored in the context of HCC, or any other solid tumors. In fact, studies evaluating dinaciclib often report its efficacy in sensitizing tumor cells to chemotherapeutic agents [69–72]. Hence, our results highlight the potential of CDK inhibitors to be paired effectively with proteasome inhibitors, specifically ixazomib, as an alternative treatment strategy for HCC.

In this study, JNK signaling activity was markedly increased following combined treatment with ixazomib and dinaciclib and played a functional role in mediating the pro-apoptotic effects of Ixa + Dina in HCC tumor cells. While a direct link between JNK activation and the anti-tumor mechanisms of either ixazomib or dinaciclib remains largely unexplored, several studies have reported the ability of these drugs to potentiate chemotherapy-associated JNK signaling. For example, the addition of ixazomib enhanced doxorubicin-induced activation of JNK and p38 in breast cancer cells [73]. Similarly, dinaciclib treatment increased vinblastine-induced acute activation of JNK-mediated apoptosis in chronic lymphocytic leukemia cells [70]. It appears that pairing both drugs in combination could have a mutual effect on JNK pathway activity in HCC. In addition, it is also likely that JNK-mediated apoptosis is not the only mechanism by which Ixa + Dina combination exert its effects in HCC tumor cells. Future investigations into other molecular changes induced by Ixa + Dina such as downregulation of DNA damage-repair pathway, as observed from our transcriptomic analysis data, could help to reveal potentially novel mechanisms that also contribute to the efficacy of Ixa + Dina in HCC.

We also note that the oncology drug screen in our study was performed at a fixed concentration for all drugs in the library, which may not be able to fully capture the effective clinical dosage of each drug. For example, the treatment concentration may be lower than the clinical dose of some drugs, resulting in the lower efficacy observed from the drug screen. While this represents a limitation for many drug screens, adjusting the treatment concentration for each individual drug is unlikely to be feasible, especially for larger drug libraries, thus screening at fixed dosages is still commonly practiced [31, 74–77]. Notably, testing a wider range of fixed concentrations during drug screening could help to better capture the drug efficacy. However, as the aim of our initial drug screen was to identify candidates that are potent against our cohort of HCC PDXOs, screening at a fixed dose was sufficiently able to highlight a distinct sensitivity of PDXOs towards proteasome inhibitors. At 1 μM, these compounds were found to have more potent cytotoxic effects against the tumor cells as compared to other drugs such as sorafenib. In addition, drugs that did not appear in the top hits such as the common HCC clinical drugs were still added into the more focused QPOP drug set, in which they were tested across a broader dose range to identify potentially effective drug combinations.

The concept of combinatorial therapy is an attractive strategy for the treatment of advanced HCC given the highly heterogeneous nature of the disease [78, 79]. The recent clinical success of atezolizumab (anti-PD-L1) and bevacizumab (anti-VEGFA) combination in prolonging progression-free survival and overall survival compared to sorafenib led to its approval by the FDA as an alternative first-line treatment for advanced HCC [3], with recommendations to potentially replace sorafenib as the new standard of care regimen [9]. With the shift in HCC treatment paradigm towards more combinatorial approaches, developing effective proteasome inhibitor-based drug combinations that can target novel cancer vulnerabilities represents a viable therapeutic strategy.

## Conclusions

Overall, we demonstrated that HCC tumors are vulnerable to proteasome inhibition and that rationally designed combination of ixazomib and dinaciclib is effective in suppressing HCC tumor growth and proliferation through mechanisms involving increased JNK activity. Our findings provide preclinical evidence for further investigation and future biomarker-based clinical evaluations of proteasome inhibitor-based drug combinations. Finally, our study highlights the feasibility of pairing rational combinatorial design approaches with patient-derived disease models for efficient development of clinically relevant combination therapies, which can also reveal potential cancer vulnerabilities for more effective targeted treatment.

## Supplementary Information

**Additional file 1: Figure S1.** Annexin V/PI assay in proteasome inhibitor-treated HCC PDXOs. (A and B) Flow cytometry analysis and corresponding quantification of annexin V/PI-stained PDXO1 (A) and PDXO11 (B) after 24-hour treatment with carfilzomib and ixazomib. Data presented as mean ± SD, *n* = 3. **, *P* < 0.01; ***, *P* < 0.001 compared to respective controls. Black and grey asterisks indicate comparison within early and late apoptosis groups, respectively. Statistical significance was determined using two-tailed Student’s *t* test. **Figure S2.** QPOP highlights Ixa+Dina as more effective than other ixazomib-based drug combinations. (A) Cluster heatmap of all two-drug combinations ranked based on QPOP output (viability_THLE2_ – viability_PDXO_). Highest rank indicates maximal difference in viability and vice versa. The corresponding two-drug combinations are listed in Table S4. (B) Polygonograms depicting QPOP-derived two-drug interaction effects on PDXO viability between ixazomib (IXA), dinaciclib (DINA), sorafenib (SORA), regorafenib (REGO), oxaliplatin (OXA), and 5-fluorouracil (5FU) in HCC PDXOs. (C) Parabolic response surface maps showing the effects of ixazomib and dinaciclib on the therapeutic output (viability_THLE2_ – viability_PDXO_) in HCC PDXOs. **Figure S3.** Annexin V/PI assay in HCC PDXOs treated with Ixa+Dina. (A and B) Analysis of Annexin V and PI-stained PDXO1 (A) and PDXO11 (B) after ixazomib and dinaciclib treatment for 24 hours. **Figure S4.** JNK pathway is involved in Ixa+Dina combined effects in HCC PDXOs. (A) Immunoblot analysis of the activation status of JNK, p38 and ERK in Ixa+Dina treated HCC PDXO1 and 11. (B) Relative mRNA levels of JUN and MAPK8 (JNK) after siRNA-mediated knockdown in Ixa+Dina treated PDXO1. Data presented as mean ± SD, *n* = 3. *, *P* < 0.05; **, *P* < 0.01. Statistical significance was determined using two-tailed Student’s *t* test. (C) Immunoblot analysis of JNK and c-Jun protein expression after siRNA transfection in Ixa+Dina treated PDXO1. (D) Immunoblots showing basal levels of JNK pathway proteins in panel of HCC PDXOs and THLE-2. **Figure S5.** Analysis of potential toxicities associated with ixazomib and dinaciclib drug treatment in vivo. (A and B) Representative H&E images of the livers, kidneys, and spleens from drug-treated PDX1 (A) and PDX11 (B) tumor-bearing mice. Scale bar = 50 μm. **Table S1.** List of primary antibodies used. **Table S2.** List of drugs and corresponding drug identification ordered by rank as displayed in Fig. 2A. **Table S3.** QPOP drug combination design (OACD) consisting of 155 combinations for nine drugs at three dosage levels (− 1, 0, 1). The nine drugs include Ixazomib (Ixa), Dinaciclib (Dina), Carfilzomib (Car), Sorafenib (Sora), Lenvatinib (Len), Regorafenib (Rego), Cabozantinib (Cabo), Oxaliplatin (Oxa), and 5-Fluorouracil (5-FU). **Table S4.** Concentrations of drugs used for QPOP on HCC PDXOs and THLE-2 at the three dosage levels (− 1, 0, 1). **Table S5.** QPOP-derived two-drug combinations as shown in Fig. S2A. Values represent the concentrations (μM) used for each drug.

## Data Availability

The data that support the findings of this study are available from the corresponding author upon reasonable request.

## Abbreviations

ATF4: Activating transcription factor 4
CDK: Cyclin-dependent kinase
CHOP: C/EBP homologous protein
CI: Combination index
DRI: Dose reduction index
ER: Endoplasmic reticulum
GADD45B: Growth arrest and DNA damage-inducible beta
GRP78: Glucose-regulated protein 78 kDa
HCC: Hepatocellular carcinoma
JNK: c-Jun N-terminal kinase
MAPK: Mitogen-activated protein kinase
OACD: Orthogonal array composite design
PARP: Poly (ADP-ribose) polymerase
PDX: Patient-derived xenograft
PDXO: PDX-derived organoids
QPOP: Quadratic phenotypic optimization platform
UPR: Unfolded protein response

## References

[1] Sung H, Ferlay J, Siegel RL, Laversanne M, Soerjomataram I, Jemal A (2021). Global cancer statistics 2020: GLOBOCAN estimates of incidence and mortality worldwide for 36 cancers in 185 countries. CA Cancer J Clin.

[2] Cheng A-L, Kang Y-K, Chen Z, Tsao C-J, Qin S, Kim JS (2009). Efficacy and safety of sorafenib in patients in the Asia-Pacific region with advanced hepatocellular carcinoma: a phase III randomised, double-blind, placebo-controlled trial. Lancet Oncol.

[3] Finn RS, Qin S, Ikeda M, Galle PR, Ducreux M, Kim T-Y (2020). Atezolizumab plus bevacizumab in unresectable hepatocellular carcinoma. N Engl J Med.

[4] Bruix J, Qin S, Merle P, Granito A, Huang Y-H, Bodoky G (2017). Regorafenib for patients with hepatocellular carcinoma who progressed on sorafenib treatment (RESORCE): a randomised, double-blind, placebo-controlled, phase 3 trial. Lancet.

[5] Kudo M, Finn RS, Qin S, Han K-H, Ikeda K, Piscaglia F (2018). Lenvatinib versus sorafenib in first-line treatment of patients with unresectable hepatocellular carcinoma: a randomised phase 3 non-inferiority trial. Lancet.

[6] Abou-Alfa GK, Meyer T, Cheng AL, El-Khoueiry AB, Rimassa L, Ryoo BY (2018). Cabozantinib in patients with advanced and progressing hepatocellular carcinoma. N Engl J Med.

[7] Zhu AX, Kang Y-K, Yen C-J, Finn RS, Galle PR, Llovet JM (2019). Ramucirumab after sorafenib in patients with advanced hepatocellular carcinoma and increased α-fetoprotein concentrations (REACH-2): a randomised, double-blind, placebo-controlled, phase 3 trial. Lancet Oncol.

[8] Vogel A, Cervantes A, Chau I, Daniele B, Llovet JM, Meyer T (2018). Hepatocellular carcinoma: ESMO clinical practice guidelines for diagnosis, treatment and follow-up. Ann Oncol.

[9] Vogel A, Martinelli E (2021). Updated treatment recommendations for hepatocellular carcinoma (HCC) from the ESMO clinical practice guidelines. Ann Oncol.

[10] Manasanch EE, Orlowski RZ (2017). Proteasome inhibitors in cancer therapy. Nat Rev Clin Oncol.

[11] Richardson PG, Sonneveld P, Schuster MW, Irwin D, Stadtmauer EA, Facon T (2005). Bortezomib or high-dose dexamethasone for relapsed multiple myeloma. N Engl J Med.

[12] Fisher RI, Bernstein SH, Kahl BS, Djulbegovic B, Robertson MJ, de Vos S (2006). Multicenter phase II study of bortezomib in patients with relapsed or refractory mantle cell lymphoma. J Clin Oncol.

[13] Goy A, Bernstein SH, Kahl BS, Djulbegovic B, Robertson MJ, de Vos S (2009). Bortezomib in patients with relapsed or refractory mantle cell lymphoma: updated time-to-event analyses of the multicenter phase 2 PINNACLE study. Ann Oncol.

[14] Niewerth D, Jansen G, Assaraf YG, Zweegman S, Kaspers GJL, Cloos J (2015). Molecular basis of resistance to proteasome inhibitors in hematological malignancies. Drug Resist Updat.

[15] Corso A, Mangiacavalli S, Varettoni M, Pascutto C, Zappasodi P, Lazzarino M (2010). Bortezomib-induced peripheral neuropathy in multiple myeloma: a comparison between previously treated and untreated patients. Leuk Res.

[16] Arastu-Kapur S, Anderl JL, Kraus M, Parlati F, Shenk KD, Lee SJ (2011). Nonproteasomal targets of the proteasome inhibitors bortezomib and carfilzomib: a link to clinical adverse events. Clin Cancer Res.

[17] Lonial S, Waller EK, Richardson PG, Jagannath S, Orlowski RZ, Giver CR (2005). Risk factors and kinetics of thrombocytopenia associated with bortezomib for relapsed, refractory multiple myeloma. Blood.

[18] Herndon TM, Deisseroth A, Kaminskas E, Kane RC, Koti KM, Rothmann MD (2013). U.S. Food and Drug Administration approval: carfilzomib for the treatment of multiple myeloma. Clin Cancer Res.

[19] Stewart AK, Rajkumar SV, Dimopoulos MA, Masszi T, Špička I, Oriol A (2014). Carfilzomib, lenalidomide, and dexamethasone for relapsed multiple myeloma. N Engl J Med.

[20] Moreau P, Masszi T, Grzasko N, Bahlis NJ, Hansson M, Pour L (2016). Oral ixazomib, lenalidomide, and dexamethasone for multiple myeloma. N Engl J Med.

[21] Roeten MSF, Cloos J, Jansen G (2018). Positioning of proteasome inhibitors in therapy of solid malignancies. Cancer Chemother Pharmacol.

[22] Aghajanian C, Soignet S, Dizon DS, Pien CS, Adams J, Elliott PJ (2002). A phase I trial of the novel proteasome inhibitor PS341 in advanced solid tumor malignancies. Clin Cancer Res.

[23] Kim GP, Mahoney MR, Szydlo D, Mok TSK, Marshke R, Holen K (2012). An international, multicenter phase II trial of bortezomib in patients with hepatocellular carcinoma. Investig New Drugs.

[24] Huang IT, Dhungel B, Shrestha R, Bridle KR, Crawford DHG, Jayachandran A (2019). Spotlight on Bortezomib: potential in the treatment of hepatocellular carcinoma. Expert Opin Investig Drugs.

[25] Augello G, Modica M, Azzolina A, Puleio R, Cassata G, Emma MR (2018). Preclinical evaluation of antitumor activity of the proteasome inhibitor MLN2238 (ixazomib) in hepatocellular carcinoma cells. Cell Death Dis.

[26] Ciombor KK, Feng Y, Benson AB, Su Y, Horton L, Short SP (2014). Phase II trial of bortezomib plus doxorubicin in hepatocellular carcinoma (E6202): a trial of the eastern cooperative oncology group. Investig New Drugs.

[27] Zhu AX, Rosmorduc O, Evans TRJ, Ross PJ, Santoro A, Carrilho FJ (2014). SEARCH: a phase III, randomized, double-blind, placebo-controlled trial of sorafenib plus erlotinib in patients with advanced hepatocellular carcinoma. J Clin Oncol.

[28] Abou-Alfa GK, Shi Q, Knox JJ, Kaubisch A, Niedzwiecki D, Posey J (2019). Assessment of treatment with sorafenib plus doxorubicin vs sorafenib alone in patients with advanced hepatocellular carcinoma: phase 3 CALGB 80802 randomized clinical trial. JAMA Oncol.

[29] Kudo M, Ueshima K, Yokosuka O, Ogasawara S, Obi S, Izumi N (2018). Sorafenib plus low-dose cisplatin and fluorouracil hepatic arterial infusion chemotherapy versus sorafenib alone in patients with advanced hepatocellular carcinoma (SILIUS): a randomised, open label, phase 3 trial. Lancet Gastroenterol Hepatol.

[30] Llovet JM, Hernandez-Gea V (2014). Hepatocellular carcinoma: reasons for phase III failure and novel perspectives on trial design. Clin Cancer Res.

[31] Rashid MBMA, Toh TB, Hooi L, Silva A, Zhang Y, Tan PF (2018). Optimizing drug combinations against multiple myeloma using a quadratic phenotypic optimization platform (QPOP). Sci Transl Med.

[32] Zarrinpar A, Lee D-K, Silva A, Datta N, Kee T, Eriksen C (2016). Individualizing liver transplant immunosuppression using a phenotypic personalized medicine platform. Sci Transl Med.

[33] Wang H, Lee DK, Chen KY, Chen JY, Zhang K, Silva A (2015). Mechanism-independent optimization of combinatorial nanodiamond and unmodified drug delivery using a phenotypically driven platform technology. ACS Nano.

[34] Blasiak A, Lim JJ, Seah SGK, Kee T, Remus A, Chye H (2021). IDentif.AI: rapidly optimizing combination therapy design against severe acute respiratory syndrome coronavirus 2 (SARS-Cov-2) with digital drug development. Bioeng Transl Med.

[35] Lee BY, Clemens DL, Silva A, Dillon BJ, Masleša-Galić S, Nava S (2017). Drug regimens identified and optimized by output-driven platform markedly reduce tuberculosis treatment time. Nat Commun.

[36] de Mel S, Rashid MBM, Zhang XY, Goh J, Lee CT, Poon LM (2020). Application of an ex-vivo drug sensitivity platform towards achieving complete remission in a refractory T-cell lymphoma. Blood Cancer J.

[37] Friedman AA, Letai A, Fisher DE, Flaherty KT (2015). Precision medicine for cancer with next-generation functional diagnostics. Nat Rev Cancer.

[38] Broutier L, Mastrogiovanni G, Verstegen MMA, Francies HE, Gavarró LM, Bradshaw CR (2017). Human primary liver cancer–derived organoid cultures for disease modeling and drug screening. Nat Med.

[39] Tentler JJ, Tan AC, Weekes CD, Jimeno A, Leong S, Pitts TM (2012). Patient-derived tumour xenografts as models for oncology drug development. Nat Rev Clin Oncol.

[40] Fong ELS, Toh TB, Lin QXX, Liu Z, Hooi L, Mohd Abdul Rashid MB (2018). Generation of matched patient-derived xenograft in vitro-in vivo models using 3D macroporous hydrogels for the study of liver cancer. Biomaterials.

[41] Broutier L, Andersson-Rolf A, Hindley CJ, Boj SF, Clevers H, Koo B-K (2016). Culture and establishment of self-renewing human and mouse adult liver and pancreas 3D organoids and their genetic manipulation. Nat Protoc.

[42] Xu H, Jaynes J, Ding X (2014). Combining two-level and three-level orthogonal arrays for factor screening and response surface exploration. Stat Sin.

[43] Chou T-C (2006). Theoretical basis, experimental design, and computerized simulation of synergism and antagonism in drug combination studies. Pharmacol Rev.

[44] Veldman-Jones MH, Brant R, Rooney C, Geh C, Emery H, Harbron CG (2015). Evaluating robustness and sensitivity of the NanoString technologies nCounter platform to enable multiplexed gene expression analysis of clinical samples. Cancer Res.

[45] Parry D, Guzi T, Shanahan F, Davis N, Prabhavalkar D, Wiswell D (2010). Dinaciclib (SCH 727965), a novel and potent cyclin-dependent kinase inhibitor. Mol Cancer Ther.

[46] Kirk CJ (2012). Discovery and development of second-generation proteasome inhibitors. Semin Hematol.

[47] Dick LR, Fleming PE (2010). Building on bortezomib: second-generation proteasome inhibitors as anti-cancer therapy. Drug Discov Today.

[48] Qin S, Bai Y, Lim HY, Thongprasert S, Chao Y, Fan J (2013). Randomized, multicenter, open-label study of oxaliplatin plus fluorouracil/leucovorin versus doxorubicin as palliative chemotherapy in patients with advanced hepatocellular carcinoma from Asia. J Clin Oncol.

[49] Qin S, Cheng Y, Liang J, Shen L, Bai Y, Li J (2014). Efficacy and safety of the FOLFOX4 regimen versus doxorubicin in Chinese patients with advanced hepatocellular carcinoma: a subgroup analysis of the EACH study. Oncologist.

[50] Lyu N, Kong Y, Mu L, Lin Y, Li J, Liu Y (2018). Hepatic arterial infusion of oxaliplatin plus fluorouracil/leucovorin vs. sorafenib for advanced hepatocellular carcinoma. J Hepatol.

[51] Lee A-H, Iwakoshi NN, Anderson KC, Glimcher LH (2003). Proteasome inhibitors disrupt the unfolded protein response in myeloma cells. Proc Natl Acad Sci U S A.

[52] Jiang H-Y, Wek RC (2005). Phosphorylation of the α-subunit of the eukaryotic initiation factor-2 (eIF2α) reduces protein synthesis and enhances apoptosis in response to proteasome inhibition. J Biol Chem.

[53] Ri M (2016). Endoplasmic-reticulum stress pathway-associated mechanisms of action of proteasome inhibitors in multiple myeloma. Int J Hematol.

[54] Obeng EA, Carlson LM, Gutman DM, Harrington WJ, Lee KP, Boise LH (2006). Proteasome inhibitors induce a terminal unfolded protein response in multiple myeloma cells. Blood.

[55] Wortel IMN, van der Meer LT, Kilberg MS, van Leeuwen FN (2017). Surviving stress: modulation of ATF4-mediated stress responses in normal and malignant cells. Trends Endocrinol Metab.

[56] Nawrocki ST, Carew JS, Pino MS, Highshaw RA, Andtbacka RHI, Dunner K (2006). Aggresome disruption: a novel strategy to enhance bortezomib-induced apoptosis in pancreatic cancer cells. Cancer Res.

[57] Kawaguchi Y, Kovacs JJ, McLaurin A, Vance JM, Ito A, Yao T-P (2003). The deacetylase HDAC6 regulates aggresome formation and cell viability in response to misfolded protein stress. Cell.

[58] Hideshima T, Bradner JE, Wong J, Chauhan D, Richardson P, Schreiber SL (2005). Small-molecule inhibition of proteasome and aggresome function induces synergistic antitumor activity in multiple myeloma. Proc Natl Acad Sci U S A.

[59] Chauhan D, Tian Z, Zhou B, Kuhn D, Orlowski R, Raje N (2011). In vitro and in vivo selective antitumor activity of a novel orally bioavailable proteasome inhibitor MLN9708 against multiple myeloma cells. Clin Cancer Res.

[60] Engür S, Dikmen M (2017). The evaluation of the anti-cancer activity of ixazomib on Caco2 colon solid tumor cells, comparison with bortezomib. Acta Clin Belg.

[61] Lin S-F, Lin J-D, Hsueh C, Chou T-C, Wong RJ (2017). A cyclin-dependent kinase inhibitor, dinaciclib in preclinical treatment models of thyroid cancer. Plos One.

[62] Hayakawa J, Mittal S, Wang Y, Korkmaz KS, Adamson E, English C (2004). Identification of promoters bound by c-Jun/ATF2 during rapid large-scale gene activation following genotoxic stress. Mol Cell.

[63] Tournier C, Hess P, Yang DD, Xu J, Turner TK, Nimnual A (2000). Requirement of JNK for stress- induced activation of the cytochrome c-mediated death pathway. Science.

[64] Dhanasekaran DN, Reddy EP (2008). JNK signaling in apoptosis. Oncogene.

[65] Dhanasekaran DN, Reddy EP (2017). JNK-signaling: a multiplexing hub in programmed cell death. Genes Cancer.

[66] Bruix J, Gores GJ, Mazzaferro V (2014). Hepatocellular carcinoma: clinical frontiers and perspectives. Gut.

[67] Huang D, Lim JQ, Cheah DMZ, Kahliab K, Laurensia Y, Pang JWL (2020). Whole-genome sequencing reveals potent therapeutic strategy for monomorphic epitheliotropic intestinal T-cell lymphoma. Blood Adv.

[68] Tang H, Xu L, Cen X, Yang L, Feng J, Li G (2020). CDK5 inhibition in vitro and in vivo induces cell death in myeloma and overcomes the obstacle of bortezomib resistance. Int J Mol Med.

[69] Saqub H, Proetsch-Gugerbauer H, Bezrookove V, Nosrati M, Vaquero EM, de Semir D (2020). Dinaciclib, a cyclin-dependent kinase inhibitor, suppresses cholangiocarcinoma growth by targeting CDK2/5/9. Sci Rep.

[70] Bates DJ, Salerni BL, Lowrey CH, Eastman A (2011). Vinblastine sensitizes leukemia cells to cyclin-dependent kinase inhibitors, inducing acute cell cycle phase-independent apoptosis. Cancer Biol Ther.

[71] Howard D, James D, Murphy K, Garcia-Parra J, Pan-Castillo B, Rex S (2021). Dinaciclib, a bimodal agent effective against endometrial cancer. Cancers (Basel).

[72] Chen Z, Wang Z, Pang JC, Yu Y, Bieerkehazhi S, Lu J (2016). Multiple CDK inhibitor dinaciclib suppresses neuroblastoma growth via inhibiting CDK2 and CDK9 activity. Sci Rep.

[73] Wang H, Yu Y, Jiang Z, Cao W-M, Wang Z, Dou J (2016). Next-generation proteasome inhibitor MLN9708 sensitizes breast cancer cells to doxorubicin-induced apoptosis. Sci Rep.

[74] Broux M, Prieto C, Demeyer S, Vanden Bempt M, Alberti-Servera L, Lodewijckx I (2019). Suz12 inactivation cooperates with JAK3 mutant signaling in the development of T-cell acute lymphoblastic leukemia. Blood.

[75] Song Y, Kim I-K, Choi I, Kim S-H, Seo HR (2018). Oxytetracycline have the therapeutic efficiency in CD133(+) HCC population through suppression CD133 expression by decreasing of protein stability of CD133. Sci Rep.

[76] Nam HJ, Kim YE, Moon B-S, Kim HY, Jung D, Choi S (2021). Azathioprine antagonizes aberrantly elevated lipid metabolism and induces apoptosis in glioblastoma. iScience.

[77] Zhan T, Ambrosi G, Wandmacher AM, Rauscher B, Betge J, Rindtorff N (2019). MEK inhibitors activate Wnt signalling and induce stem cell plasticity in colorectal cancer. Nat Commun.

[78] Zhang Q, Lou Y, Yang J, Wang J, Feng J, Zhao Y (2019). Integrated multiomic analysis reveals comprehensive tumour heterogeneity and novel immunophenotypic classification in hepatocellular carcinomas. Gut.

[79] Ding X, He M, Chan AWH, Song QX, Sze SC, Chen H (2019). Genomic and epigenomic features of primary and recurrent hepatocellular carcinomas. Gastroenterology.

