# Supplementary material for: Rational drug combination design in patient-derived avatars reveals effective inhibition of hepatocellular carcinoma with proteasome and CDK inhibitors
Source: J Exp Clin Cancer Res. 2022 Aug 15;41:249. doi: 10.1186/s13046-022-02436-9 (PMC9377092; doi:10.1186/s13046-022-02436-9)
Supplement: Supplementary file 1 — Additional file 1: Figure S1. Annexin V/PI assay in proteasome inhibitor-treated HCC PDXOs. (A and B) Flow cytometry analysis and corresponding quantification of annexin V/PI-stained PDXO1 (A) and PDXO11 (B) after 24-hour treatment with carfilzomib and ixazomib. Data presented as mean ± SD, n = 3. **, P < 0.01; ***, P < 0.001 compared to respective controls. Black and grey asterisks indicate comparison within early and late apoptosis groups, respectively. Statistical significance was determined using two-tailed Student’s t test. Figure S2. QPOP highlights Ixa+Dina as more effective than other ixazomib-based drug combinations. (A) Cluster heatmap of all two-drug combinations ranked based on QPOP output (viabilityTHLE2 – viabilityPDXO). Highest rank indicates maximal difference in viability and vice versa. The corresponding two-drug combinations are listed in Table S4. (B) Polygonograms depicting QPOP-derived two-drug interaction effects on PDXO viability between ixazomib (IXA), dinaciclib (DINA), sorafenib (SORA), regorafenib (REGO), oxaliplatin (OXA), and 5-fluorouracil (5FU) in HCC PDXOs. (C) Parabolic response surface maps showing the effects of ixazomib and dinaciclib on the therapeutic output (viabilityTHLE2 – viabilityPDXO) in HCC PDXOs. Figure S3. Annexin V/PI assay in HCC PDXOs treated with Ixa+Dina. (A and B) Analysis of Annexin V and PI-stained PDXO1 (A) and PDXO11 (B) after ixazomib and dinaciclib treatment for 24 hours. Figure S4. JNK pathway is involved in Ixa+Dina combined effects in HCC PDXOs. (A) Immunoblot analysis of the activation status of JNK, p38 and ERK in Ixa+Dina treated HCC PDXO1 and 11. (B) Relative mRNA levels of JUN and MAPK8 (JNK) after siRNA-mediated knockdown in Ixa+Dina treated PDXO1. Data presented as mean ± SD, n = 3. *, P < 0.05; **, P < 0.01. Statistical significance was determined using two-tailed Student’s t test. (C) Immunoblot analysis of JNK and c-Jun protein expression after siRNA transfection in Ixa+Dina treated PDXO1. (D) [file 13046_2022_2436_MOESM1_ESM.docx]

**Supplemental Material**


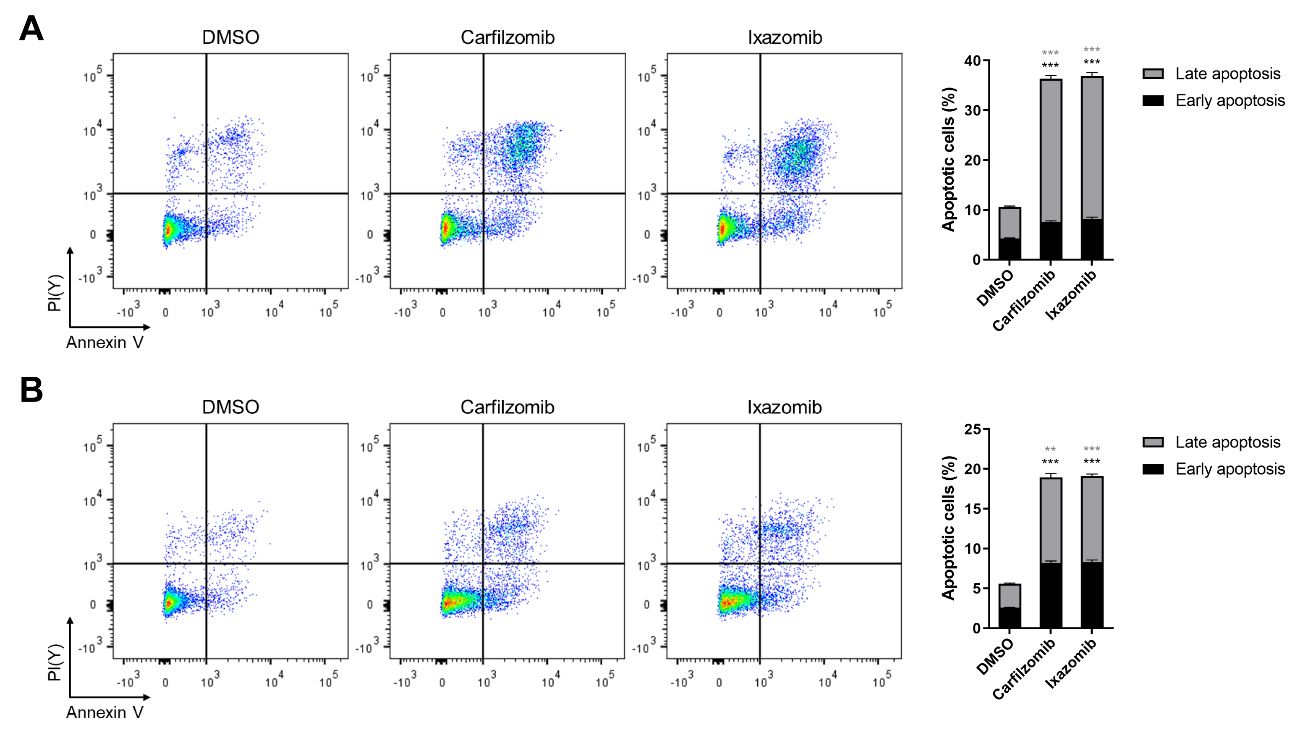


**Figure S1. Annexin V/PI assay in proteasome inhibitor-treated HCC PDXOs. (A and B)** Flow cytometry analysis and corresponding quantification of annexin V/PI-stained PDXO1 (A) and PDXO11 (B) after 24-hour treatment with carfilzomib and ixazomib. Data presented as mean ± SD, n = 3. **, *P* < 0.01; ***, *P* < 0.001 compared to respective controls. Black and grey asterisks indicate comparison within early and late apoptosis groups, respectively. Statistical significance was determined using two-tailed Student’s *t* test.


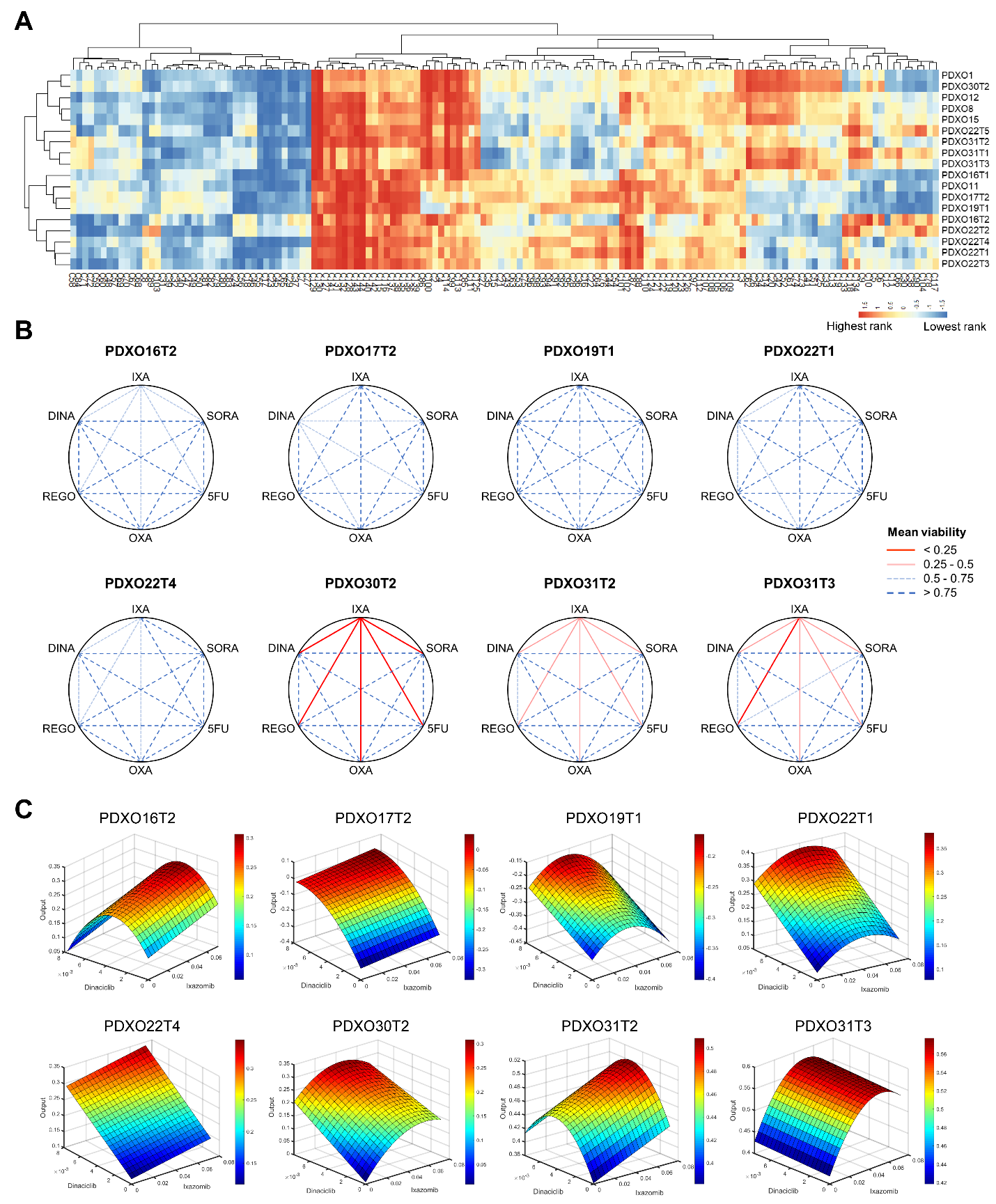


**Figure S2. QPOP highlights Ixa+Dina as more effective than other ixazomib-based drug combinations**. **(A)** Cluster heatmap of all two-drug combinations ranked based on QPOP output (viability_THLE2_ – viability_PDXO_). Highest rank indicates maximal difference in viability and vice versa. The corresponding two-drug combinations are listed in Table S4. **(B)** Polygonograms depicting QPOP-derived two-drug interaction effects on PDXO viability between ixazomib (IXA), dinaciclib (DINA), sorafenib (SORA), regorafenib (REGO), oxaliplatin (OXA), and 5-fluorouracil (5FU) in HCC PDXOs. **(C)** Parabolic response surface maps showing the effects of ixazomib and dinaciclib on the therapeutic output (viability_THLE2_ – viability_PDXO_) in HCC PDXOs.


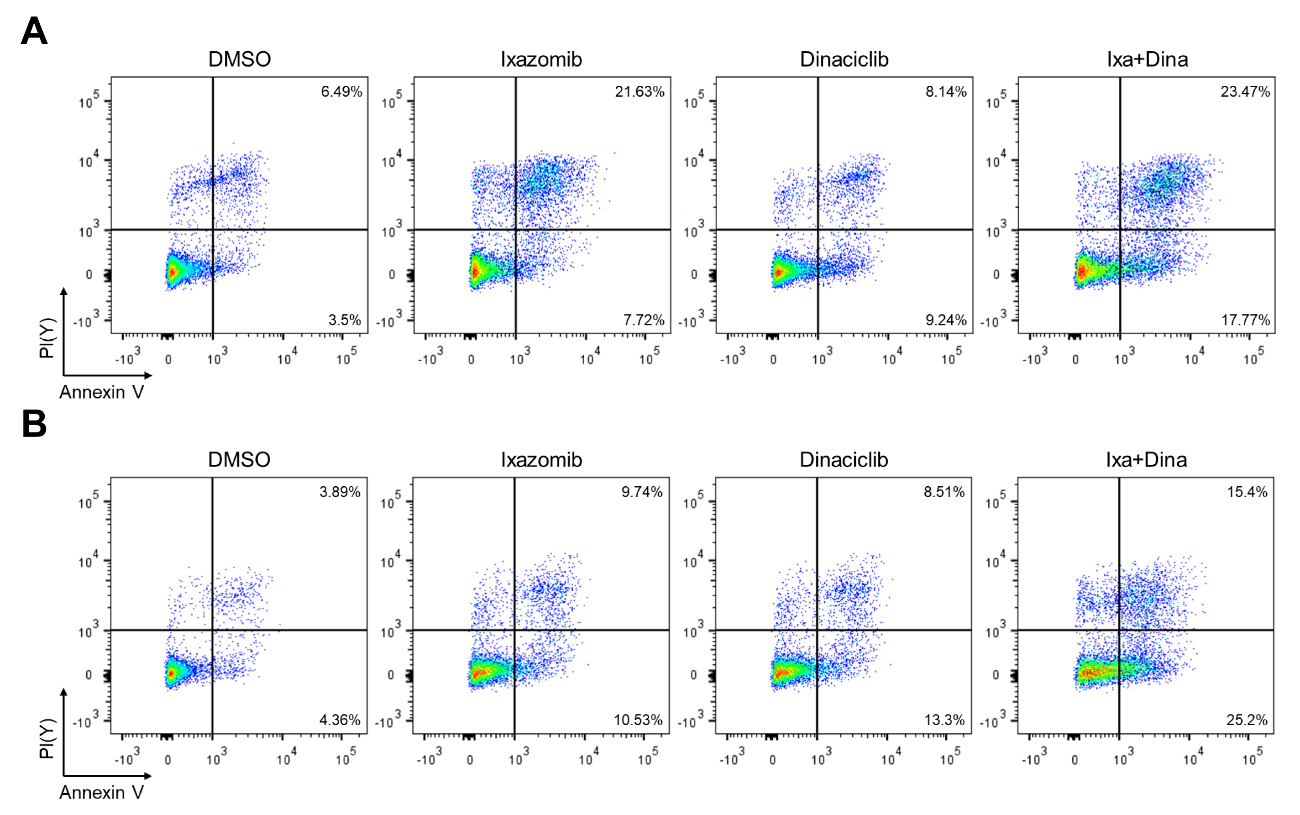


**Figure S3. Annexin V/PI assay in HCC PDXOs treated with Ixa+Dina. (A and B)** Analysis of Annexin V and PI-stained PDXO1 (A) and PDXO11 (B) after ixazomib and dinaciclib treatment for 24 hours.


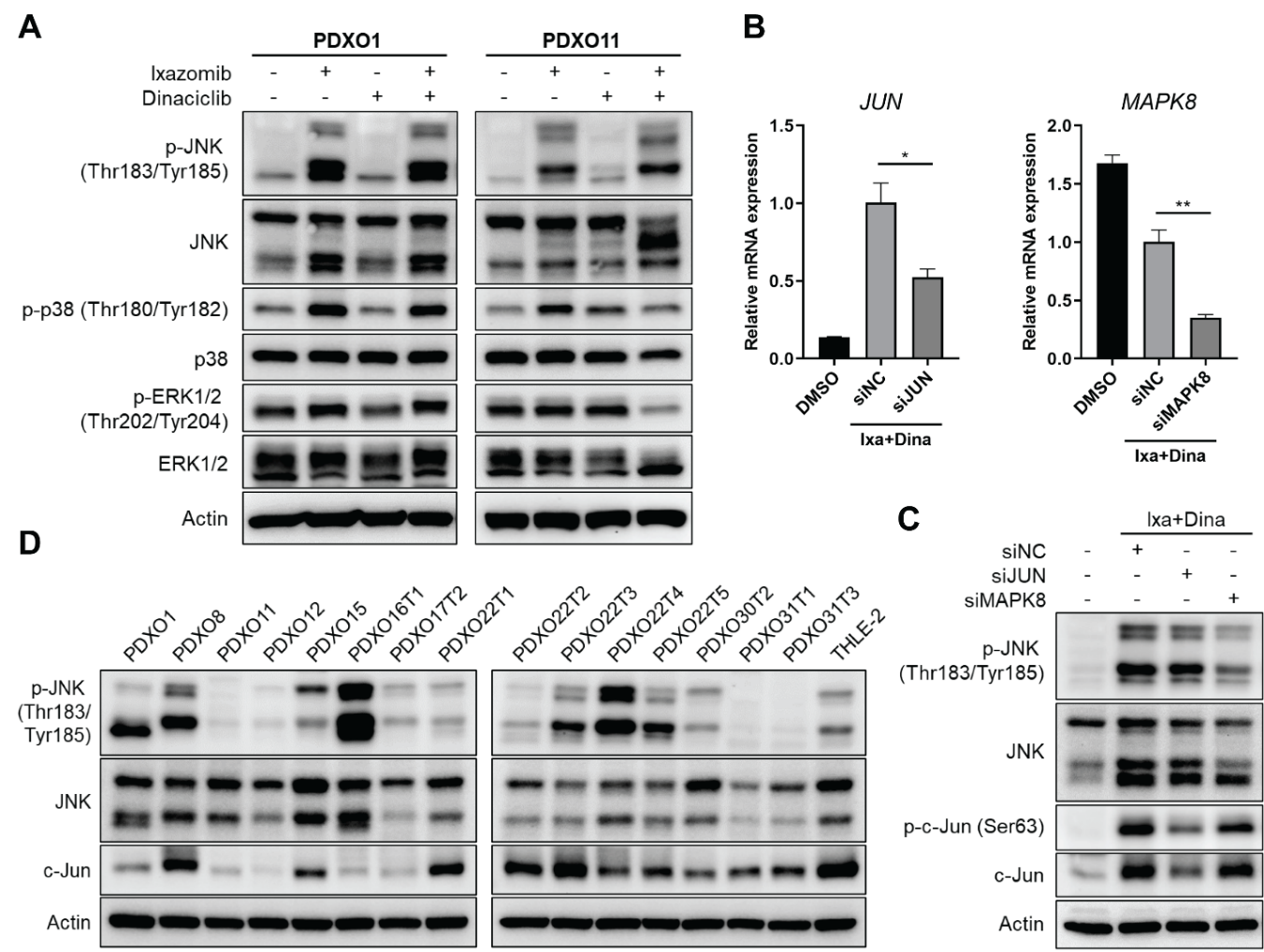


**Figure S4. JNK pathway is involved in Ixa+Dina combined effects in HCC PDXOs. (A)** Immunoblot analysis of the activation status of JNK, p38 and ERK in Ixa+Dina treated HCC PDXO1 and 11. **(B)** Relative mRNA levels of JUN and MAPK8 (JNK) after siRNA-mediated knockdown in Ixa+Dina treated PDXO1. Data presented as mean ± SD, n = 3. *, *P* < 0.05; **, *P* < 0.01. Statistical significance was determined using two-tailed Student’s *t* test. **(C)** Immunoblot analysis of JNK and c-Jun protein expression after siRNA transfection in Ixa+Dina treated PDXO1. **(D)** Immunoblots showing basal levels of JNK pathway proteins in panel of HCC PDXOs and THLE-2.


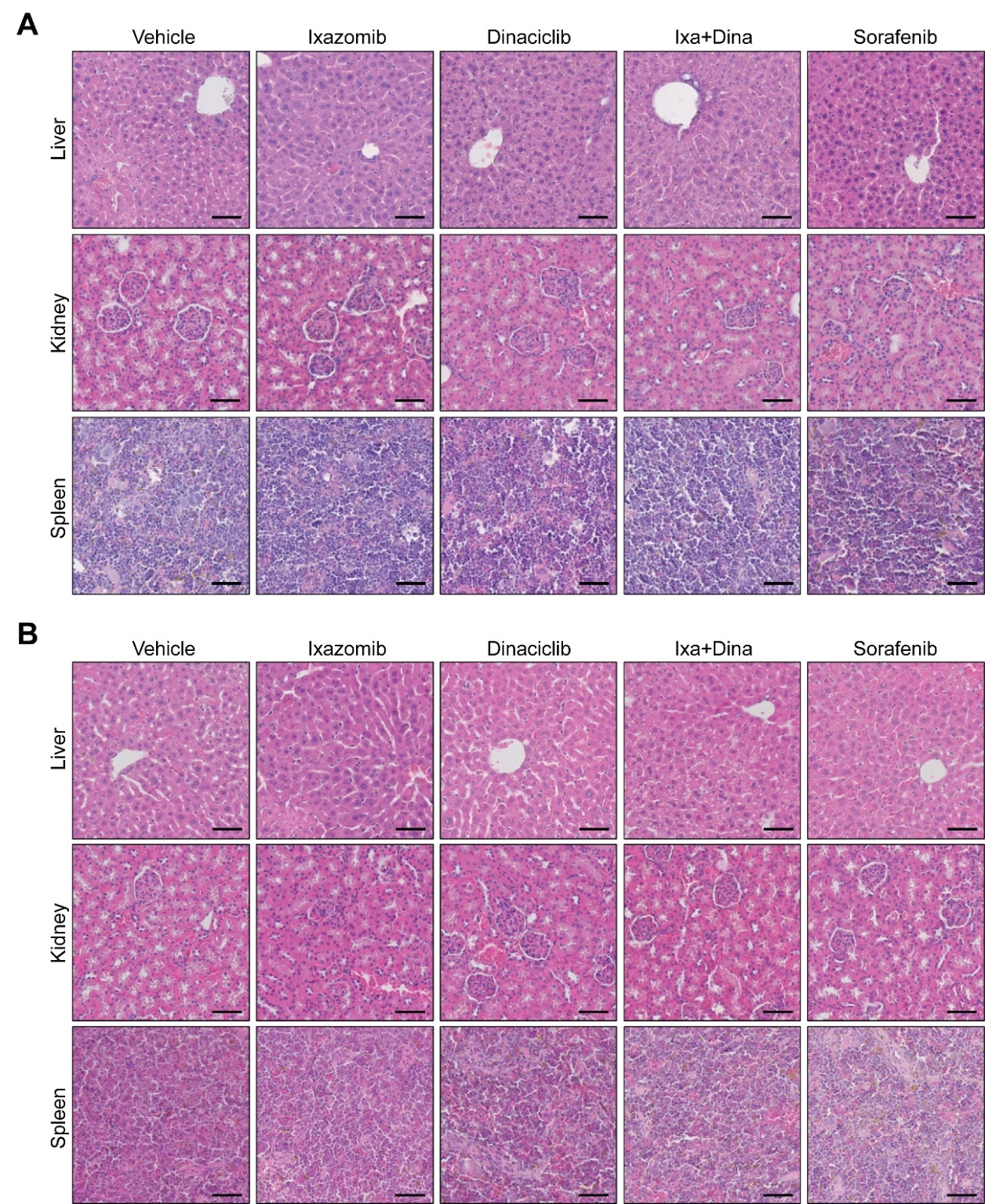


**Figure S5. Analysis of potential toxicities associated with ixazomib and dinaciclib drug treatment *in vivo*.** **(A and B)** Representative H&E images of the livers, kidneys, and spleens from drug-treated PDX1 (A) and PDX11 (B) tumor-bearing mice. Scale bar = 50 μm.

Table S1. List of primary antibodies used.

| **Name** | **Supplier** | **Cat no.** | **Clone no.** | **Dilution** |
| --- | --- | --- | --- | --- |
| AFP | Abcam | ab133617 | EPAFP61 | 1:250 |
| HepPar1 | NovusBio | NBP2-45272 | - | 1:400 |
| CK19 | Abcam | ab52625 | EP1580Y | 1:500 |
| GPC3 | Abcam | ab66596 | - | 1:100 |
| CHOP | Cell Signaling | 2895 | L63F7 | 1:1000 |
| GRP78/BiP | Cell Signaling | 3177 | C50B12 | 1:1000 |
| ATF4 | Santa Cruz | sc-390063 | B-3 | 1:400 |
| Cleaved Caspase 3 (Asp175) | Cell Signaling | 9664 | 5A1E | 1:1000 |
| Caspase 3 | Cell Signaling | 9662 | - | 1:1000 |
| PARP | Cell Signaling | 9542 | - | 1:1000 |
| c-Jun | Cell Signaling | 9165 | 60A8 | 1:1000 |
| Phospho-c-Jun (Ser63) | Cell Signaling | 9261 | - | 1:1000 |
| JNK | Cell Signaling | 9252 | - | 1:1000 |
| Phospho-JNK (Thr183/Tyr185) | Cell Signaling | 4668 | 81E11 | 1:1000 |
| Phospho-Rb (Ser807/811) | Cell Signaling | 8516 | D20B12 | 1:1000 |
| CDK1/cdc2 | Cell Signaling | 9116 | POH1 | 1:1000 |
| CDK2 | Cell Signaling | 2546 | 78B2 | 1:1000 |
| CDK5 | Invitrogen | AHZ0492 | DC34 | 1:500 |
| CDK9 | Cell Signaling | 2316 | C12F7 | 1:1000 |
| NF-κB1 p105/p50 | Cell Signaling | 13586 | D4P4D | 1:1000 |
| ERK1/2 | Cell Signaling | 4695 | 137F5 | 1:2000 |
| Phospho-ERK1/2 (Thr202/Tyr204) | Cell Signaling | 4370 | D13.14.4E | 1:1000 |
| p38 | Cell Signaling | 9212 | - | 1:1000 |
| Phospho-p38 (Thr180/Tyr182) | Cell Signaling | 4511 | D3F9 | 1:1000 |
| β-Actin | Sigma-Aldrich | A5441 | AC-15 | 1:10,000 |
| Ki67 | Abcam | ab92742 | EPR3610 | 1:500 |

Table S2. List of drugs and corresponding drug identification ordered by rank as displayed in Figure 2A.

| **Rank** | **Drug ID** | **Drug name** |
| --- | --- | --- |
| 1 | 34 | Bortezomib |
| 2 | 45 | Carfilzomib |
| 3 | 142 | Ixazomib |
| 4 | 78 | Dinaciclib |
| 5 | 179 | Mubritinib |
| 6 | 206 | Ponatinib |
| 7 | 57 | Cobimetinib |
| 8 | 221 | Romidepsin |
| 9 | 128 | GSK2126458 |
| 10 | 260 | Vinblastine |
| 11 | 192 | Pacritinib |
| 12 | 73 | Daunorubicin |
| 13 | 261 | Vinblastine sulfate |
| 14 | 58 | Colchicine |
| 15 | 118 | Ganetespib |
| 16 | 131 | Idarubicin HCl |
| 17 | 203 | Pimasertib |
| 18 | 263 | Vinorelbine |
| 19 | 145 | LDK378 |
| 20 | 79 | Disulfiram |
| 21 | 2 | 17-AAG |
| 22 | 262 | Vincristine |
| 23 | 25 | Bardoxolone methyl |
| 24 | 245 | Tivantinib |
| 25 | 220 | Rigosertib |
| 26 | 182 | Napabucasin |
| 27 | 178 | Mocetinostat |
| 28 | 241 | Temsirolimus |
| 29 | 9 | Afatinib |
| 30 | 251 | Trametinib |
| 31 | 207 | Poziotinib |
| 32 | 91 | Entrectinib |
| 33 | 188 | Oltipraz |
| 34 | 216 | Rapamycin |
| 35 | 60 | CUDC-101 |
| 36 | 121 | Gefitinib |
| 37 | 62 | CX-4945 (Silmitasertib) |
| 38 | 7 | ABT-263 (Navitoclax) |
| 39 | 37 | Cabazitaxel |
| 40 | 208 | Pracinostat |
| 41 | 143 | Lapatinib |
| 42 | 99 | Everolimus |
| 43 | 122 | Geniposidic acid |
| 44 | 217 | Regorafenib |
| 45 | 69 | Dacomitinib |
| 46 | 152 | Linsitinib |
| 47 | 5 | 5-Methoxypsoralen |
| 48 | 127 | GS-9973 |
| 49 | 120 | GDC-0941 |
| 50 | 254 | Triciribine |
| 51 | 93 | Epirubicin HCl |
| 52 | 201 | Pexidartinib |
| 53 | 135 | INCB-024360 |
| 54 | 249 | Topotecan HCl |
| 55 | 90 | Entinostat |
| 56 | 165 | MEK162 |
| 57 | 141 | Ispinesib |
| 58 | 194 | Panobinostat |
| 59 | 197 | PCI-24781 |
| 60 | 20 | Axitinib |
| 61 | 42 | Canagliflozin |
| 62 | 211 | Quizartinib |
| 63 | 156 | LY2157299 |
| 64 | 94 | Epothilone B |
| 65 | 126 | GM 6001 |
| 66 | 32 | Bleomycin sulfate |
| 67 | 72 | Dasatinib |
| 68 | 115 | Fosbretabulin disodium |
| 69 | 89 | EMD-1214063 |
| 70 | 10 | Afatinib dimaleate |
| 71 | 40 | CAL-101 (Idelalisib) |
| 72 | 22 | AZD6244 (Selumetinib) |
| 73 | 219 | Ricolinostat |
| 74 | 233 | Sorafenib tosylate |
| 75 | 229 | Silymarin |
| 76 | 84 | Doxorubicin HCl |
| 77 | 23 | AZD-9291 |
| 78 | 176 | Mitoxantrone HCl |
| 79 | 47 | Carmustine |
| 80 | 266 | YM155 |
| 81 | 56 | CO-1686 |
| 82 | 193 | Palbociclib isethionate |
| 83 | 210 | Quercetin |
| 84 | 133 | Imatinib |
| 85 | 50 | Chlorambucil |
| 86 | 196 | Pazopanib HCl |
| 87 | 223 | Rosiglitazone |
| 88 | 33 | BMS-708163 |
| 89 | 114 | Foretinib |
| 90 | 111 | Fluorouracil |
| 91 | 218 | Regorafenib HCl |
| 92 | 144 | LDE225 (Erismodegib) |
| 93 | 66 | Cytidine |
| 94 | 61 | Curcumin |
| 95 | 139 | Irinotecan HCl trihydrate |
| 96 | 185 | Nilotinib |
| 97 | 181 | Mycophenolic acid |
| 98 | 18 | Apatinib |
| 99 | 4 | 5-Azacytidine |
| 100 | 155 | Lonidamine |
| 101 | 88 | Embelin |
| 102 | 160 | Maraviroc |
| 103 | 54 | Cisplatin |
| 104 | 95 | Erlotinib HCl |
| 105 | 264 | Vorinostat |
| 106 | 186 | Nintedanib |
| 107 | 46 | Carmofur |
| 108 | 41 | Calcitriol |
| 109 | 82 | Doxercalciferol |
| 110 | 237 | Sunitinib malate |
| 111 | 137 | IPI-145 |
| 112 | 59 | Crenolanib |
| 113 | 257 | Vandetanib |
| 114 | 238 | Tamibarotene |
| 115 | 236 | Sunitinib |
| 116 | 129 | Hydrocortisone |
| 117 | 119 | GDC-0449 (Vismodegib) |
| 118 | 199 | Pelitinib |
| 119 | 35 | Bosutinib |
| 120 | 154 | Lonafarnib |
| 121 | 29 | BI6727 (Volasertib) |
| 122 | 228 | Saracatinib |
| 123 | 11 | Alendronate |
| 124 | 202 | Phenformin HCl |
| 125 | 12 | Aminoglutethimide |
| 126 | 191 | Paclitaxel |
| 127 | 130 | Hydroxyurea |
| 128 | 239 | Tamoxifen citrate |
| 129 | 17 | Anastrozole |
| 130 | 80 | Docetaxel |
| 131 | 157 | LY2228820 |
| 132 | 171 | Mevastatin |
| 133 | 31 | BIRB 796 |
| 134 | 65 | Cyclosporin A |
| 135 | 243 | TG101348 |
| 136 | 28 | Bexarotene |
| 137 | 161 | Masitinib |
| 138 | 169 | Metformin HCl |
| 139 | 71 | DAPT (GSI-IX) |
| 140 | 225 | Ruxolitinib |
| 141 | 83 | Doxifluridine |
| 142 | 81 | Dovitinib |
| 143 | 19 | Aprepitant |
| 144 | 107 | Fingolimod |
| 145 | 26 | Belinostat |
| 146 | 76 | Dexamethasone acetate |
| 147 | 52 | Cimetidine |
| 148 | 51 | Cilengitide |
| 149 | 151 | Linifanib |
| 150 | 163 | Medroxyprogesterone acetate |
| 151 | 39 | Cabozantinib malate |
| 152 | 117 | Fulvestrant |
| 153 | 70 | Dapagliflozin |
| 154 | 44 | Capsaicin |
| 155 | 242 | Teniposide |
| 156 | 38 | Cabozantinib |
| 157 | 140 | Isotretinoin |
| 158 | 153 | Lomustine |
| 159 | 86 | Elacridar |
| 160 | 96 | Estradiol |
| 161 | 215 | Ranolazine |
| 162 | 158 | LY2784544 |
| 163 | 55 | Clofarabine |
| 164 | 110 | Fludarabine phosphate |
| 165 | 103 | Fasudil HCl |
| 166 | 21 | Azathioprine |
| 167 | 67 | Dabrafenib |
| 168 | 167 | Mercaptopurine (6-MP) |
| 169 | 138 | Irinotecan |
| 170 | 212 | Raloxifene HCl |
| 171 | 195 | Pazopanib |
| 172 | 30 | Bicalutamide |
| 173 | 227 | SAR245409 |
| 174 | 166 | Melphalan |
| 175 | 248 | TOK-001 |
| 176 | 36 | Busulfan |
| 177 | 198 | PCI-32765 (Ibrutinib) |
| 178 | 183 | Nedaplatin |
| 179 | 101 | Exemestane |
| 180 | 234 | Sotrastaurin |
| 181 | 252 | Tretinoin |
| 182 | 232 | Sorafenib |
| 183 | 92 | Enzastaurin |
| 184 | 1 | 10-DAB |
| 185 | 226 | Salirasib |
| 186 | 268 | Zileuton |
| 187 | 149 | Lenvatinib |
| 188 | 204 | Pioglitazone |
| 189 | 267 | Zibotentan |
| 190 | 8 | ABT-888 (Veliparib) |
| 191 | 104 | Febuxostat |
| 192 | 259 | Vemurafenib |
| 193 | 48 | Cediranib |
| 194 | 231 | Sodium phenylbutyrate |
| 195 | 235 | Streptozocin |
| 196 | 43 | Capecitabine |
| 197 | 125 | GLPG0634 |
| 198 | 68 | Dacarbazine |
| 199 | 136 | Iniparib |
| 200 | 87 | Ellagic acid |
| 201 | 174 | Mitomycin C |
| 202 | 240 | Temozolomide |
| 203 | 14 | Amonafide |
| 204 | 97 | Estrone |
| 205 | 63 | Cyclophosphamide |
| 206 | 112 | Flutamide |
| 207 | 224 | Rucaparib |
| 208 | 230 | Simvastatin |
| 209 | 159 | Malotilate |
| 210 | 147 | Leflunomide |
| 211 | 108 | Floxuridine |
| 212 | 109 | Fludarabine |
| 213 | 116 | FT-207 |
| 214 | 250 | Toremifene citrate |
| 215 | 256 | Sodium valproate |
| 216 | 222 | Roscovitine |
| 217 | 175 | Mitotane |
| 218 | 209 | Pralatrexate |
| 219 | 75 | Dexamethasone |
| 220 | 189 | Orotic acid |
| 221 | 187 | Olaparib |
| 222 | 246 | Tivozanib |
| 223 | 190 | Oxaliplatin |
| 224 | 105 | FG-4592 |
| 225 | 27 | Bendamustine HCl |
| 226 | 168 | Mesna |
| 227 | 247 | Tofacitinib |
| 228 | 184 | Nelarabine |
| 229 | 162 | MDV3100 (Enzalutamide) |
| 230 | 106 | Finasteride |
| 231 | 213 | Raltitrexed |
| 232 | 146 | LEE011 |
| 233 | 53 | Ciprofibrate |
| 234 | 124 | Gimeracil |
| 235 | 177 | MLN8237 (Alisertib) |
| 236 | 255 | Trifluridine |
| 237 | 113 | Fluvastatin sodium |
| 238 | 150 | Letrozole |
| 239 | 77 | Dimesna |
| 240 | 64 | Cyclophosphamide monohydrate |
| 241 | 132 | Ifosfamide |
| 242 | 15 | Amuvatinib |
| 243 | 265 | VX-680 (Tozasertib) |
| 244 | 170 | Methotrexate |
| 245 | 172 | Mifepristone |
| 246 | 148 | Lenalidomide |
| 247 | 98 | Etoposide |
| 248 | 13 | Amisulpride |
| 249 | 3 | 2-Methoxyestradiol |
| 250 | 123 | Genistein |
| 251 | 85 | Dutasteride |
| 252 | 74 | Decitabine |
| 253 | 100 | EX 527 |
| 254 | 16 | Anagrelide HCl |
| 255 | 214 | Ramelteon |
| 256 | 200 | Pemetrexed |
| 257 | 258 | Vatalanib 2HCl |
| 258 | 253 | Triamcinolone acetonide |
| 259 | 244 | Thalidomide |
| 260 | 180 | Mycophenolate mofetil |
| 261 | 173 | Miltefosine |
| 262 | 102 | Ezetimibe |
| 263 | 49 | Celecoxib |
| 264 | 164 | Megestrol acetate |
| 265 | 134 | Imatinib mesylate |
| 266 | 24 | Barasertib |
| 267 | 205 | Pomalidomide |
| 268 | 6 | Abiraterone |

Table S3. QPOP drug combination design (OACD) consisting of 155 combinations for nine drugs at three dosage levels (-1, 0, 1). The nine drugs include Ixazomib (Ixa), Dinaciclib (Dina), Carfilzomib (Car), Sorafenib (Sora), Lenvatinib (Len), Regorafenib (Rego), Cabozantinib (Cabo), Oxaliplatin (Oxa), and 5-Fluorouracil (5-FU).

| **Combo** | **Ixa** | **Dina** | **Car** | **Sora** | **Len** | **Rego** | **Cabo** | **Oxa** | **5-FU** |
| --- | --- | --- | --- | --- | --- | --- | --- | --- | --- |
| 1 | -1 | -1 | -1 | -1 | -1 | -1 | -1 | -1 | -1 |
| 2 | -1 | -1 | -1 | -1 | -1 | -1 | 1 | -1 | 1 |
| 3 | -1 | -1 | -1 | -1 | -1 | 1 | -1 | -1 | 1 |
| 4 | -1 | -1 | -1 | -1 | -1 | 1 | 1 | -1 | -1 |
| 5 | -1 | -1 | -1 | -1 | 1 | -1 | -1 | 1 | -1 |
| 6 | -1 | -1 | -1 | -1 | 1 | -1 | 1 | 1 | 1 |
| 7 | -1 | -1 | -1 | -1 | 1 | 1 | -1 | 1 | 1 |
| 8 | -1 | -1 | -1 | -1 | 1 | 1 | 1 | 1 | -1 |
| 9 | -1 | -1 | -1 | 1 | -1 | -1 | -1 | 1 | -1 |
| 10 | -1 | -1 | -1 | 1 | -1 | -1 | 1 | 1 | 1 |
| 11 | -1 | -1 | -1 | 1 | -1 | 1 | -1 | 1 | 1 |
| 12 | -1 | -1 | -1 | 1 | -1 | 1 | 1 | 1 | -1 |
| 13 | -1 | -1 | -1 | 1 | 1 | -1 | -1 | -1 | -1 |
| 14 | -1 | -1 | -1 | 1 | 1 | -1 | 1 | -1 | 1 |
| 15 | -1 | -1 | -1 | 1 | 1 | 1 | -1 | -1 | 1 |
| 16 | -1 | -1 | -1 | 1 | 1 | 1 | 1 | -1 | -1 |
| 17 | -1 | -1 | 1 | -1 | -1 | -1 | -1 | 1 | 1 |
| 18 | -1 | -1 | 1 | -1 | -1 | -1 | 1 | 1 | -1 |
| 19 | -1 | -1 | 1 | -1 | -1 | 1 | -1 | 1 | -1 |
| 20 | -1 | -1 | 1 | -1 | -1 | 1 | 1 | 1 | 1 |
| 21 | -1 | -1 | 1 | -1 | 1 | -1 | -1 | -1 | 1 |
| 22 | -1 | -1 | 1 | -1 | 1 | -1 | 1 | -1 | -1 |
| 23 | -1 | -1 | 1 | -1 | 1 | 1 | -1 | -1 | -1 |
| 24 | -1 | -1 | 1 | -1 | 1 | 1 | 1 | -1 | 1 |
| 25 | -1 | -1 | 1 | 1 | -1 | -1 | -1 | -1 | 1 |
| 26 | -1 | -1 | 1 | 1 | -1 | -1 | 1 | -1 | -1 |
| 27 | -1 | -1 | 1 | 1 | -1 | 1 | -1 | -1 | -1 |
| 28 | -1 | -1 | 1 | 1 | -1 | 1 | 1 | -1 | 1 |
| 29 | -1 | -1 | 1 | 1 | 1 | -1 | -1 | 1 | 1 |
| 30 | -1 | -1 | 1 | 1 | 1 | -1 | 1 | 1 | -1 |
| 31 | -1 | -1 | 1 | 1 | 1 | 1 | -1 | 1 | -1 |
| 32 | -1 | -1 | 1 | 1 | 1 | 1 | 1 | 1 | 1 |
| 33 | -1 | 1 | -1 | -1 | -1 | -1 | -1 | 1 | 1 |
| 34 | -1 | 1 | -1 | -1 | -1 | -1 | 1 | 1 | -1 |
| 35 | -1 | 1 | -1 | -1 | -1 | 1 | -1 | 1 | -1 |
| 36 | -1 | 1 | -1 | -1 | -1 | 1 | 1 | 1 | 1 |
| 37 | -1 | 1 | -1 | -1 | 1 | -1 | -1 | -1 | 1 |
| 38 | -1 | 1 | -1 | -1 | 1 | -1 | 1 | -1 | -1 |
| 39 | -1 | 1 | -1 | -1 | 1 | 1 | -1 | -1 | -1 |
| 40 | -1 | 1 | -1 | -1 | 1 | 1 | 1 | -1 | 1 |
| 41 | -1 | 1 | -1 | 1 | -1 | -1 | -1 | -1 | 1 |
| 42 | -1 | 1 | -1 | 1 | -1 | -1 | 1 | -1 | -1 |
| 43 | -1 | 1 | -1 | 1 | -1 | 1 | -1 | -1 | -1 |
| 44 | -1 | 1 | -1 | 1 | -1 | 1 | 1 | -1 | 1 |
| 45 | -1 | 1 | -1 | 1 | 1 | -1 | -1 | 1 | 1 |
| 46 | -1 | 1 | -1 | 1 | 1 | -1 | 1 | 1 | -1 |
| 47 | -1 | 1 | -1 | 1 | 1 | 1 | -1 | 1 | -1 |
| 48 | -1 | 1 | -1 | 1 | 1 | 1 | 1 | 1 | 1 |
| 49 | -1 | 1 | 1 | -1 | -1 | -1 | -1 | -1 | -1 |
| 50 | -1 | 1 | 1 | -1 | -1 | -1 | 1 | -1 | 1 |
| 51 | -1 | 1 | 1 | -1 | -1 | 1 | -1 | -1 | 1 |
| 52 | -1 | 1 | 1 | -1 | -1 | 1 | 1 | -1 | -1 |
| 53 | -1 | 1 | 1 | -1 | 1 | -1 | -1 | 1 | -1 |
| 54 | -1 | 1 | 1 | -1 | 1 | -1 | 1 | 1 | 1 |
| 55 | -1 | 1 | 1 | -1 | 1 | 1 | -1 | 1 | 1 |
| 56 | -1 | 1 | 1 | -1 | 1 | 1 | 1 | 1 | -1 |
| 57 | -1 | 1 | 1 | 1 | -1 | -1 | -1 | 1 | -1 |
| 58 | -1 | 1 | 1 | 1 | -1 | -1 | 1 | 1 | 1 |
| 59 | -1 | 1 | 1 | 1 | -1 | 1 | -1 | 1 | 1 |
| 60 | -1 | 1 | 1 | 1 | -1 | 1 | 1 | 1 | -1 |
| 61 | -1 | 1 | 1 | 1 | 1 | -1 | -1 | -1 | -1 |
| 62 | -1 | 1 | 1 | 1 | 1 | -1 | 1 | -1 | 1 |
| 63 | -1 | 1 | 1 | 1 | 1 | 1 | -1 | -1 | 1 |
| 64 | -1 | 1 | 1 | 1 | 1 | 1 | 1 | -1 | -1 |
| 65 | 1 | -1 | -1 | -1 | -1 | -1 | -1 | 1 | 1 |
| 66 | 1 | -1 | -1 | -1 | -1 | -1 | 1 | 1 | -1 |
| 67 | 1 | -1 | -1 | -1 | -1 | 1 | -1 | 1 | -1 |
| 68 | 1 | -1 | -1 | -1 | -1 | 1 | 1 | 1 | 1 |
| 69 | 1 | -1 | -1 | -1 | 1 | -1 | -1 | -1 | 1 |
| 70 | 1 | -1 | -1 | -1 | 1 | -1 | 1 | -1 | -1 |
| 71 | 1 | -1 | -1 | -1 | 1 | 1 | -1 | -1 | -1 |
| 72 | 1 | -1 | -1 | -1 | 1 | 1 | 1 | -1 | 1 |
| 73 | 1 | -1 | -1 | 1 | -1 | -1 | -1 | -1 | 1 |
| 74 | 1 | -1 | -1 | 1 | -1 | -1 | 1 | -1 | -1 |
| 75 | 1 | -1 | -1 | 1 | -1 | 1 | -1 | -1 | -1 |
| 76 | 1 | -1 | -1 | 1 | -1 | 1 | 1 | -1 | 1 |
| 77 | 1 | -1 | -1 | 1 | 1 | -1 | -1 | 1 | 1 |
| 78 | 1 | -1 | -1 | 1 | 1 | -1 | 1 | 1 | -1 |
| 79 | 1 | -1 | -1 | 1 | 1 | 1 | -1 | 1 | -1 |
| 80 | 1 | -1 | -1 | 1 | 1 | 1 | 1 | 1 | 1 |
| 81 | 1 | -1 | 1 | -1 | -1 | -1 | -1 | -1 | -1 |
| 82 | 1 | -1 | 1 | -1 | -1 | -1 | 1 | -1 | 1 |
| 83 | 1 | -1 | 1 | -1 | -1 | 1 | -1 | -1 | 1 |
| 84 | 1 | -1 | 1 | -1 | -1 | 1 | 1 | -1 | -1 |
| 85 | 1 | -1 | 1 | -1 | 1 | -1 | -1 | 1 | -1 |
| 86 | 1 | -1 | 1 | -1 | 1 | -1 | 1 | 1 | 1 |
| 87 | 1 | -1 | 1 | -1 | 1 | 1 | -1 | 1 | 1 |
| 88 | 1 | -1 | 1 | -1 | 1 | 1 | 1 | 1 | -1 |
| 89 | 1 | -1 | 1 | 1 | -1 | -1 | -1 | 1 | -1 |
| 90 | 1 | -1 | 1 | 1 | -1 | -1 | 1 | 1 | 1 |
| 91 | 1 | -1 | 1 | 1 | -1 | 1 | -1 | 1 | 1 |
| 92 | 1 | -1 | 1 | 1 | -1 | 1 | 1 | 1 | -1 |
| 93 | 1 | -1 | 1 | 1 | 1 | -1 | -1 | -1 | -1 |
| 94 | 1 | -1 | 1 | 1 | 1 | -1 | 1 | -1 | 1 |
| 95 | 1 | -1 | 1 | 1 | 1 | 1 | -1 | -1 | 1 |
| 96 | 1 | -1 | 1 | 1 | 1 | 1 | 1 | -1 | -1 |
| 97 | 1 | 1 | -1 | -1 | -1 | -1 | -1 | -1 | -1 |
| 98 | 1 | 1 | -1 | -1 | -1 | -1 | 1 | -1 | 1 |
| 99 | 1 | 1 | -1 | -1 | -1 | 1 | -1 | -1 | 1 |
| 100 | 1 | 1 | -1 | -1 | -1 | 1 | 1 | -1 | -1 |
| 101 | 1 | 1 | -1 | -1 | 1 | -1 | -1 | 1 | -1 |
| 102 | 1 | 1 | -1 | -1 | 1 | -1 | 1 | 1 | 1 |
| 103 | 1 | 1 | -1 | -1 | 1 | 1 | -1 | 1 | 1 |
| 104 | 1 | 1 | -1 | -1 | 1 | 1 | 1 | 1 | -1 |
| 105 | 1 | 1 | -1 | 1 | -1 | -1 | -1 | 1 | -1 |
| 106 | 1 | 1 | -1 | 1 | -1 | -1 | 1 | 1 | 1 |
| 107 | 1 | 1 | -1 | 1 | -1 | 1 | -1 | 1 | 1 |
| 108 | 1 | 1 | -1 | 1 | -1 | 1 | 1 | 1 | -1 |
| 109 | 1 | 1 | -1 | 1 | 1 | -1 | -1 | -1 | -1 |
| 110 | 1 | 1 | -1 | 1 | 1 | -1 | 1 | -1 | 1 |
| 111 | 1 | 1 | -1 | 1 | 1 | 1 | -1 | -1 | 1 |
| 112 | 1 | 1 | -1 | 1 | 1 | 1 | 1 | -1 | -1 |
| 113 | 1 | 1 | 1 | -1 | -1 | -1 | -1 | 1 | 1 |
| 114 | 1 | 1 | 1 | -1 | -1 | -1 | 1 | 1 | -1 |
| 115 | 1 | 1 | 1 | -1 | -1 | 1 | -1 | 1 | -1 |
| 116 | 1 | 1 | 1 | -1 | -1 | 1 | 1 | 1 | 1 |
| 117 | 1 | 1 | 1 | -1 | 1 | -1 | -1 | -1 | 1 |
| 118 | 1 | 1 | 1 | -1 | 1 | -1 | 1 | -1 | -1 |
| 119 | 1 | 1 | 1 | -1 | 1 | 1 | -1 | -1 | -1 |
| 120 | 1 | 1 | 1 | -1 | 1 | 1 | 1 | -1 | 1 |
| 121 | 1 | 1 | 1 | 1 | -1 | -1 | -1 | -1 | 1 |
| 122 | 1 | 1 | 1 | 1 | -1 | -1 | 1 | -1 | -1 |
| 123 | 1 | 1 | 1 | 1 | -1 | 1 | -1 | -1 | -1 |
| 124 | 1 | 1 | 1 | 1 | -1 | 1 | 1 | -1 | 1 |
| 125 | 1 | 1 | 1 | 1 | 1 | -1 | -1 | 1 | 1 |
| 126 | 1 | 1 | 1 | 1 | 1 | -1 | 1 | 1 | -1 |
| 127 | 1 | 1 | 1 | 1 | 1 | 1 | -1 | 1 | -1 |
| 128 | 1 | 1 | 1 | 1 | 1 | 1 | 1 | 1 | 1 |
| 129 | -1 | -1 | -1 | -1 | -1 | -1 | -1 | -1 | -1 |
| 130 | -1 | -1 | 0 | 0 | -1 | 1 | 0 | 1 | 1 |
| 131 | -1 | -1 | 1 | 1 | -1 | 0 | 1 | 0 | 0 |
| 132 | -1 | 0 | -1 | 0 | 1 | 0 | -1 | 0 | 1 |
| 133 | -1 | 0 | 0 | 1 | 1 | -1 | 0 | -1 | 0 |
| 134 | -1 | 0 | 1 | -1 | 1 | 1 | 1 | 1 | -1 |
| 135 | -1 | 1 | -1 | 1 | 0 | 1 | -1 | 1 | 0 |
| 136 | -1 | 1 | 0 | -1 | 0 | 0 | 0 | 0 | -1 |
| 137 | -1 | 1 | 1 | 0 | 0 | -1 | 1 | -1 | 1 |
| 138 | 0 | -1 | -1 | 0 | 0 | 0 | 0 | -1 | 0 |
| 139 | 0 | -1 | 0 | 1 | 0 | -1 | 1 | 1 | -1 |
| 140 | 0 | -1 | 1 | -1 | 0 | 1 | -1 | 0 | 1 |
| 141 | 0 | 0 | -1 | 1 | -1 | 1 | 0 | 0 | -1 |
| 142 | 0 | 0 | 0 | -1 | -1 | 0 | 1 | -1 | 1 |
| 143 | 0 | 0 | 1 | 0 | -1 | -1 | -1 | 1 | 0 |
| 144 | 0 | 1 | -1 | -1 | 1 | -1 | 0 | 1 | 1 |
| 145 | 0 | 1 | 0 | 0 | 1 | 1 | 1 | 0 | 0 |
| 146 | 0 | 1 | 1 | 1 | 1 | 0 | -1 | -1 | -1 |
| 147 | 1 | -1 | -1 | 1 | 1 | 1 | 1 | -1 | 1 |
| 148 | 1 | -1 | 0 | -1 | 1 | 0 | -1 | 1 | 0 |
| 149 | 1 | -1 | 1 | 0 | 1 | -1 | 0 | 0 | -1 |
| 150 | 1 | 0 | -1 | -1 | 0 | -1 | 1 | 0 | 0 |
| 151 | 1 | 0 | 0 | 0 | 0 | 1 | -1 | -1 | -1 |
| 152 | 1 | 0 | 1 | 1 | 0 | 0 | 0 | 1 | 1 |
| 153 | 1 | 1 | -1 | 0 | -1 | 0 | 1 | 1 | -1 |
| 154 | 1 | 1 | 0 | 1 | -1 | -1 | -1 | 0 | 1 |
| 155 | 1 | 1 | 1 | -1 | -1 | 1 | 0 | -1 | 0 |

Table S4. Concentrations of drugs used for QPOP on HCC PDXOs and THLE-2 at the three dosage levels (-1, 0, 1).

| **Drug** | **-1** | **0** | **1** |
| --- | --- | --- | --- |
|  | **IC_0_ (µM)** | **IC_10_ (µM)** | **IC_20_ (µM)** |
| Ixazomib | 0 | 0.036 | 0.072 |
| Dinaciclib | 0 | 0.00386 | 0.00772 |
| Carfilzomib | 0 | 0.0658 | 0.1316 |
| Sorafenib | 0 | 1.476 | 2.952 |
| Lenvatinib | 0 | 0.6 | 1.2 |
| Regorafenib | 0 | 2.218 | 4.436 |
| Cabozantinib | 0 | 1 | 2 |
| Oxaliplatin | 0 | 4 | 8 |
| 5-FU | 0 | 16 | 32 |

Table S5. QPOP-derived two-drug combinations as shown in Figure S2A. Values represent the concentrations (μM) used for each drug.

| **Combo** | **Ixa** | **Dina** | **Car** | **Sora** | **Len** | **Rego** | **Cabo** | **Oxa** | **5-FU** |
| --- | --- | --- | --- | --- | --- | --- | --- | --- | --- |
| C1 | 0.036 | 0.00386 | 0 | 0 | 0 | 0 | 0 | 0 | 0 |
| C2 | 0.072 | 0.00386 | 0 | 0 | 0 | 0 | 0 | 0 | 0 |
| C3 | 0.036 | 0.00772 | 0 | 0 | 0 | 0 | 0 | 0 | 0 |
| C4 | 0.072 | 0.00772 | 0 | 0 | 0 | 0 | 0 | 0 | 0 |
| C5 | 0.036 | 0 | 0.0658 | 0 | 0 | 0 | 0 | 0 | 0 |
| C6 | 0.072 | 0 | 0.0658 | 0 | 0 | 0 | 0 | 0 | 0 |
| C7 | 0 | 0.00386 | 0.0658 | 0 | 0 | 0 | 0 | 0 | 0 |
| C8 | 0 | 0.00772 | 0.0658 | 0 | 0 | 0 | 0 | 0 | 0 |
| C9 | 0.036 | 0 | 0.1316 | 0 | 0 | 0 | 0 | 0 | 0 |
| C10 | 0.072 | 0 | 0.1316 | 0 | 0 | 0 | 0 | 0 | 0 |
| C11 | 0 | 0.00386 | 0.1316 | 0 | 0 | 0 | 0 | 0 | 0 |
| C12 | 0 | 0.00772 | 0.1316 | 0 | 0 | 0 | 0 | 0 | 0 |
| C13 | 0.036 | 0 | 0 | 1.476 | 0 | 0 | 0 | 0 | 0 |
| C14 | 0.072 | 0 | 0 | 1.476 | 0 | 0 | 0 | 0 | 0 |
| C15 | 0 | 0.00386 | 0 | 1.476 | 0 | 0 | 0 | 0 | 0 |
| C16 | 0 | 0.00772 | 0 | 1.476 | 0 | 0 | 0 | 0 | 0 |
| C17 | 0 | 0 | 0.0658 | 1.476 | 0 | 0 | 0 | 0 | 0 |
| C18 | 0 | 0 | 0.1316 | 1.476 | 0 | 0 | 0 | 0 | 0 |
| C19 | 0.036 | 0 | 0 | 2.952 | 0 | 0 | 0 | 0 | 0 |
| C20 | 0.072 | 0 | 0 | 2.952 | 0 | 0 | 0 | 0 | 0 |
| C21 | 0 | 0.00386 | 0 | 2.952 | 0 | 0 | 0 | 0 | 0 |
| C22 | 0 | 0.00772 | 0 | 2.952 | 0 | 0 | 0 | 0 | 0 |
| C23 | 0 | 0 | 0.0658 | 2.952 | 0 | 0 | 0 | 0 | 0 |
| C24 | 0 | 0 | 0.1316 | 2.952 | 0 | 0 | 0 | 0 | 0 |
| C25 | 0.036 | 0 | 0 | 0 | 0.6 | 0 | 0 | 0 | 0 |
| C26 | 0.072 | 0 | 0 | 0 | 0.6 | 0 | 0 | 0 | 0 |
| C27 | 0 | 0.00386 | 0 | 0 | 0.6 | 0 | 0 | 0 | 0 |
| C28 | 0 | 0.00772 | 0 | 0 | 0.6 | 0 | 0 | 0 | 0 |
| C29 | 0 | 0 | 0.0658 | 0 | 0.6 | 0 | 0 | 0 | 0 |
| C30 | 0 | 0 | 0.1316 | 0 | 0.6 | 0 | 0 | 0 | 0 |
| C31 | 0 | 0 | 0 | 1.476 | 0.6 | 0 | 0 | 0 | 0 |
| C32 | 0 | 0 | 0 | 2.952 | 0.6 | 0 | 0 | 0 | 0 |
| C33 | 0.036 | 0 | 0 | 0 | 1.2 | 0 | 0 | 0 | 0 |
| C34 | 0.072 | 0 | 0 | 0 | 1.2 | 0 | 0 | 0 | 0 |
| C35 | 0 | 0.00386 | 0 | 0 | 1.2 | 0 | 0 | 0 | 0 |
| C36 | 0 | 0.00772 | 0 | 0 | 1.2 | 0 | 0 | 0 | 0 |
| C37 | 0 | 0 | 0.0658 | 0 | 1.2 | 0 | 0 | 0 | 0 |
| C38 | 0 | 0 | 0.1316 | 0 | 1.2 | 0 | 0 | 0 | 0 |
| C39 | 0 | 0 | 0 | 1.476 | 1.2 | 0 | 0 | 0 | 0 |
| C40 | 0 | 0 | 0 | 2.952 | 1.2 | 0 | 0 | 0 | 0 |
| C41 | 0.036 | 0 | 0 | 0 | 0 | 2.218 | 0 | 0 | 0 |
| C42 | 0.072 | 0 | 0 | 0 | 0 | 2.218 | 0 | 0 | 0 |
| C43 | 0 | 0.00386 | 0 | 0 | 0 | 2.218 | 0 | 0 | 0 |
| C44 | 0 | 0.00772 | 0 | 0 | 0 | 2.218 | 0 | 0 | 0 |
| C45 | 0 | 0 | 0.0658 | 0 | 0 | 2.218 | 0 | 0 | 0 |
| C46 | 0 | 0 | 0.1316 | 0 | 0 | 2.218 | 0 | 0 | 0 |
| C47 | 0 | 0 | 0 | 1.476 | 0 | 2.218 | 0 | 0 | 0 |
| C48 | 0 | 0 | 0 | 2.952 | 0 | 2.218 | 0 | 0 | 0 |
| C49 | 0 | 0 | 0 | 0 | 0.6 | 2.218 | 0 | 0 | 0 |
| C50 | 0 | 0 | 0 | 0 | 1.2 | 2.218 | 0 | 0 | 0 |
| C51 | 0.036 | 0 | 0 | 0 | 0 | 4.436 | 0 | 0 | 0 |
| C52 | 0.072 | 0 | 0 | 0 | 0 | 4.436 | 0 | 0 | 0 |
| C53 | 0 | 0.00386 | 0 | 0 | 0 | 4.436 | 0 | 0 | 0 |
| C54 | 0 | 0.00772 | 0 | 0 | 0 | 4.436 | 0 | 0 | 0 |
| C55 | 0 | 0 | 0.0658 | 0 | 0 | 4.436 | 0 | 0 | 0 |
| C56 | 0 | 0 | 0.1316 | 0 | 0 | 4.436 | 0 | 0 | 0 |
| C57 | 0 | 0 | 0 | 1.476 | 0 | 4.436 | 0 | 0 | 0 |
| C58 | 0 | 0 | 0 | 2.952 | 0 | 4.436 | 0 | 0 | 0 |
| C59 | 0 | 0 | 0 | 0 | 0.6 | 4.436 | 0 | 0 | 0 |
| C60 | 0 | 0 | 0 | 0 | 1.2 | 4.436 | 0 | 0 | 0 |
| C61 | 0.036 | 0 | 0 | 0 | 0 | 0 | 1 | 0 | 0 |
| C62 | 0.072 | 0 | 0 | 0 | 0 | 0 | 1 | 0 | 0 |
| C63 | 0 | 0.00386 | 0 | 0 | 0 | 0 | 1 | 0 | 0 |
| C64 | 0 | 0.00772 | 0 | 0 | 0 | 0 | 1 | 0 | 0 |
| C65 | 0 | 0 | 0.0658 | 0 | 0 | 0 | 1 | 0 | 0 |
| C66 | 0 | 0 | 0.1316 | 0 | 0 | 0 | 1 | 0 | 0 |
| C67 | 0 | 0 | 0 | 1.476 | 0 | 0 | 1 | 0 | 0 |
| C68 | 0 | 0 | 0 | 2.952 | 0 | 0 | 1 | 0 | 0 |
| C69 | 0 | 0 | 0 | 0 | 0.6 | 0 | 1 | 0 | 0 |
| C70 | 0 | 0 | 0 | 0 | 1.2 | 0 | 1 | 0 | 0 |
| C71 | 0 | 0 | 0 | 0 | 0 | 2.218 | 1 | 0 | 0 |
| C72 | 0 | 0 | 0 | 0 | 0 | 4.436 | 1 | 0 | 0 |
| C73 | 0.036 | 0 | 0 | 0 | 0 | 0 | 2 | 0 | 0 |
| C74 | 0.072 | 0 | 0 | 0 | 0 | 0 | 2 | 0 | 0 |
| C75 | 0 | 0.00386 | 0 | 0 | 0 | 0 | 2 | 0 | 0 |
| C76 | 0 | 0.00772 | 0 | 0 | 0 | 0 | 2 | 0 | 0 |
| C77 | 0 | 0 | 0.0658 | 0 | 0 | 0 | 2 | 0 | 0 |
| C78 | 0 | 0 | 0.1316 | 0 | 0 | 0 | 2 | 0 | 0 |
| C79 | 0 | 0 | 0 | 1.476 | 0 | 0 | 2 | 0 | 0 |
| C80 | 0 | 0 | 0 | 2.952 | 0 | 0 | 2 | 0 | 0 |
| C81 | 0 | 0 | 0 | 0 | 0.6 | 0 | 2 | 0 | 0 |
| C82 | 0 | 0 | 0 | 0 | 1.2 | 0 | 2 | 0 | 0 |
| C83 | 0 | 0 | 0 | 0 | 0 | 2.218 | 2 | 0 | 0 |
| C84 | 0 | 0 | 0 | 0 | 0 | 4.436 | 2 | 0 | 0 |
| C85 | 0.036 | 0 | 0 | 0 | 0 | 0 | 0 | 4 | 0 |
| C86 | 0.072 | 0 | 0 | 0 | 0 | 0 | 0 | 4 | 0 |
| C87 | 0 | 0.00386 | 0 | 0 | 0 | 0 | 0 | 4 | 0 |
| C88 | 0 | 0.00772 | 0 | 0 | 0 | 0 | 0 | 4 | 0 |
| C89 | 0 | 0 | 0.0658 | 0 | 0 | 0 | 0 | 4 | 0 |
| C90 | 0 | 0 | 0.1316 | 0 | 0 | 0 | 0 | 4 | 0 |
| C91 | 0 | 0 | 0 | 1.476 | 0 | 0 | 0 | 4 | 0 |
| C92 | 0 | 0 | 0 | 2.952 | 0 | 0 | 0 | 4 | 0 |
| C93 | 0 | 0 | 0 | 0 | 0.6 | 0 | 0 | 4 | 0 |
| C94 | 0 | 0 | 0 | 0 | 1.2 | 0 | 0 | 4 | 0 |
| C95 | 0 | 0 | 0 | 0 | 0 | 2.218 | 0 | 4 | 0 |
| C96 | 0 | 0 | 0 | 0 | 0 | 4.436 | 0 | 4 | 0 |
| C97 | 0 | 0 | 0 | 0 | 0 | 0 | 1 | 4 | 0 |
| C98 | 0 | 0 | 0 | 0 | 0 | 0 | 2 | 4 | 0 |
| C99 | 0.036 | 0 | 0 | 0 | 0 | 0 | 0 | 8 | 0 |
| C100 | 0.072 | 0 | 0 | 0 | 0 | 0 | 0 | 8 | 0 |
| C101 | 0 | 0.00386 | 0 | 0 | 0 | 0 | 0 | 8 | 0 |
| C102 | 0 | 0.00772 | 0 | 0 | 0 | 0 | 0 | 8 | 0 |
| C103 | 0 | 0 | 0.0658 | 0 | 0 | 0 | 0 | 8 | 0 |
| C104 | 0 | 0 | 0.1316 | 0 | 0 | 0 | 0 | 8 | 0 |
| C105 | 0 | 0 | 0 | 1.476 | 0 | 0 | 0 | 8 | 0 |
| C106 | 0 | 0 | 0 | 2.952 | 0 | 0 | 0 | 8 | 0 |
| C107 | 0 | 0 | 0 | 0 | 0.6 | 0 | 0 | 8 | 0 |
| C108 | 0 | 0 | 0 | 0 | 1.2 | 0 | 0 | 8 | 0 |
| C109 | 0 | 0 | 0 | 0 | 0 | 2.218 | 0 | 8 | 0 |
| C110 | 0 | 0 | 0 | 0 | 0 | 4.436 | 0 | 8 | 0 |
| C111 | 0 | 0 | 0 | 0 | 0 | 0 | 1 | 8 | 0 |
| C112 | 0 | 0 | 0 | 0 | 0 | 0 | 2 | 8 | 0 |
| C113 | 0.036 | 0 | 0 | 0 | 0 | 0 | 0 | 0 | 16 |
| C114 | 0.072 | 0 | 0 | 0 | 0 | 0 | 0 | 0 | 16 |
| C115 | 0 | 0.00386 | 0 | 0 | 0 | 0 | 0 | 0 | 16 |
| C116 | 0 | 0.00772 | 0 | 0 | 0 | 0 | 0 | 0 | 16 |
| C117 | 0 | 0 | 0.0658 | 0 | 0 | 0 | 0 | 0 | 16 |
| C118 | 0 | 0 | 0.1316 | 0 | 0 | 0 | 0 | 0 | 16 |
| C119 | 0 | 0 | 0 | 1.476 | 0 | 0 | 0 | 0 | 16 |
| C120 | 0 | 0 | 0 | 2.952 | 0 | 0 | 0 | 0 | 16 |
| C121 | 0 | 0 | 0 | 0 | 0.6 | 0 | 0 | 0 | 16 |
| C122 | 0 | 0 | 0 | 0 | 1.2 | 0 | 0 | 0 | 16 |
| C123 | 0 | 0 | 0 | 0 | 0 | 2.218 | 0 | 0 | 16 |
| C124 | 0 | 0 | 0 | 0 | 0 | 4.436 | 0 | 0 | 16 |
| C125 | 0 | 0 | 0 | 0 | 0 | 0 | 1 | 0 | 16 |
| C126 | 0 | 0 | 0 | 0 | 0 | 0 | 2 | 0 | 16 |
| C127 | 0 | 0 | 0 | 0 | 0 | 0 | 0 | 4 | 16 |
| C128 | 0 | 0 | 0 | 0 | 0 | 0 | 0 | 8 | 16 |
| C129 | 0.036 | 0 | 0 | 0 | 0 | 0 | 0 | 0 | 32 |
| C130 | 0.072 | 0 | 0 | 0 | 0 | 0 | 0 | 0 | 32 |
| C131 | 0 | 0.00386 | 0 | 0 | 0 | 0 | 0 | 0 | 32 |
| C132 | 0 | 0.00772 | 0 | 0 | 0 | 0 | 0 | 0 | 32 |
| C133 | 0 | 0 | 0.0658 | 0 | 0 | 0 | 0 | 0 | 32 |
| C134 | 0 | 0 | 0.1316 | 0 | 0 | 0 | 0 | 0 | 32 |
| C135 | 0 | 0 | 0 | 1.476 | 0 | 0 | 0 | 0 | 32 |
| C136 | 0 | 0 | 0 | 2.952 | 0 | 0 | 0 | 0 | 32 |
| C137 | 0 | 0 | 0 | 0 | 0.6 | 0 | 0 | 0 | 32 |
| C138 | 0 | 0 | 0 | 0 | 1.2 | 0 | 0 | 0 | 32 |
| C139 | 0 | 0 | 0 | 0 | 0 | 2.218 | 0 | 0 | 32 |
| C140 | 0 | 0 | 0 | 0 | 0 | 4.436 | 0 | 0 | 32 |
| C141 | 0 | 0 | 0 | 0 | 0 | 0 | 1 | 0 | 32 |
| C142 | 0 | 0 | 0 | 0 | 0 | 0 | 2 | 0 | 32 |
| C143 | 0 | 0 | 0 | 0 | 0 | 0 | 0 | 4 | 32 |
| C144 | 0 | 0 | 0 | 0 | 0 | 0 | 0 | 8 | 32 |
